# Roles of adenine methylation in the physiology of *Lacticaseibacillus paracasei*

**DOI:** 10.1038/s41467-023-38291-1

**Published:** 2023-05-06

**Authors:** Jie Zhao, Meng Zhang, Wenyan Hui, Yue Zhang, Jing Wang, Shaojing Wang, Lai-Yu Kwok, Jian Kong, Heping Zhang, Wenyi Zhang

**Affiliations:** 1 Key Laboratory of Dairy Biotechnology and Engineering, Ministry of Education, Inner Mongolia Agricultural University, Hohhot, 010018 China; 2 Key Laboratory of Dairy Products Processing, Ministry of Agriculture and Rural Affairs, Inner Mongolia Agricultural University, Hohhot, 010018 China; 3 Inner Mongolia Key Laboratory of Dairy Biotechnology and Engineering, Inner Mongolia Agricultural University, Hohhot, 010018 China; 4 Key Laboratory of Molecular Microbiology and Technology, Ministry of Education, College of Life Sciences, Nankai University, Tianjin, 300071 China; 5 State Key Laboratory of Microbial Technology, Shandong University, Qingdao, 266237 China

**Keywords:** Food microbiology, Applied microbiology, Bacterial genomics, DNA methylation

## Abstract

*Lacticaseibacillus paracasei* is an economically important bacterial species, used in the food industry and as a probiotic. Here, we investigate the roles of N6-methyladenine (6mA) modification in *L. paracasei* using multi-omics and high-throughput chromosome conformation capture (Hi-C) analyses. The distribution of 6mA-modified sites varies across the genomes of 28 strains, and appears to be enriched near genes involved in carbohydrate metabolism. A *pglX* mutant, defective in 6mA modification, shows transcriptomic alterations but only modest changes in growth and genomic spatial organization.

## Abstract (web summary)

The bacterium *Lacticaseibacillus paracasei* is used in the food industry and as a probiotic. Here, the authors use multi-omics and high-throughput chromosome conformation capture analyses to investigate the roles of a type of DNA methylation (N6-methyladenine modification) in this organism.

## Introduction

In bacteria, DNA methylation is a universal epigenetic mechanism achieved by transferring a methyl group onto specific positions of cytosine and adenine to form 5-methylcytosine, 4-methylcytosine, or 6-methyladenine (6mA)^1^. DNA methylation is associated with important physiological functions, including chromosome replication, genome stability, correction of DNA mismatches, and cell cycle-coupled transcription^2^. In earlier high-throughput functional genomics studies, bacterial DNA methylation is largely analyzed by using the single-molecule real-time (SMRT) sequencing technology that produces genome-level methylation profiles directly without DNA pretreatment^3^. Nowadays, an increasing number of studies have applied an integrated approach by combining other omics and high-throughput technologies, like transcriptomics, proteomics, and high-throughput chromosome conformation capture (Hi-C), to reveal potential associations or even interactions between methylated sites and gene expression^4^. Data obtained by such an approach provide useful information for decoding the underlying molecular mechanisms of how DNA methylation regulates cellular and physiological functions.

6mA is the most prevalent among the three common types of base methylation patterns identified in bacteria. It plays roles in regulating gene expression and forming persister cells in pathogenic bacteria^5, 6^. The cellular function of 6mA is far less known in non-pathogens like lactobacilli, which are commonly found and indeed often used in food fermentation^7,8^. A recent metagenomics study has linked the plasmids and phages to their respective host genomes based on plasmid-borne DNA methylation motifs to reveal biologically relevant insights^9^. Species-/strain-specificity of DNA methylation has been demonstrated in the species *Lacticaseibacillus* *paracasei* and *Lactiplantibacillus plantarum*^10^. Moreover, DNA methylation could influence the transformation efficiency of *L. paracasei* hosts^11^.

Although high-throughput sequencing-based methylomics studies have started to reveal information like methylation profiles and features in certain non-pathogens like lactobacilli, such knowledge remains at a phenotypic level without providing much functional and mechanistic data. Moreover, the inter- and intraspecies distribution of 6mA at the population level remains unexplored, and the interactions between 6mA and metabolic capacity are also largely unknown in lactobacilli. One well-studied species among lactobacilli is *L. paracasei*, which is an economically important species in the food industry^12^. Under specific industrial fermentation processes, they have to survive harsh conditions with limited nutrient supply for an extended period of time, when they continue to contribute to flavor development in the food matrix^13,14^. Development and exploitation of *L. paracasei* strains have mainly relied on screening according to their genetic and/or genomic backgrounds^15^.

Based on the premise that epigenetic codes hidden in the genome also involve in regulating the physiological behavior of the host, this study aimed to perform a systematic investigation on *L. paracasei* by a combined use of genomics, methylomics, transcriptomics, proteomics, Hi-C, and metabolomics analyses. The biological insights obtained through omics and high-throughput data integration shed light on the molecular and functional roles of epigenetic modifications, which would promote novel, cutting-edge frontier research in the field of probiotics. Meanwhile, the current work has paved a new way to improve the production characteristics of lactobacilli.

## Results

### Genome sequencing, annotation, and phylogenetic reconstruction of *L. paracasei* isolates

This study sequenced and constructed a total of 27 closed genomes (average genome coverage of 257- to 643-fold) of *L. paracasei* by the combined use of Illumina and SMRT sequencing. For comparison, *L. paracasei* Zhang previously sequenced by our research team was also added to the present data set^16^.

**Fig. 1:**
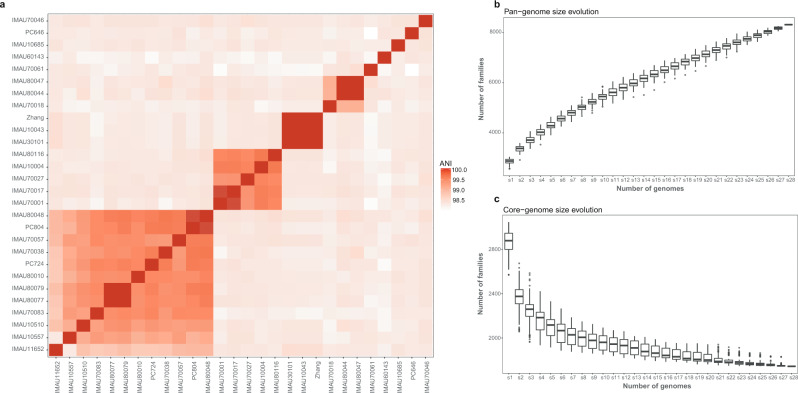
Intraspecific genome similarity, core- and pan-genomes of *Lacticaseibacillusparacasei*. Heatmap displaying whole-genome level average nucleotide identity (ANI) across 27 *L. paracasei* isolates and *L. paracasei* Zhang (**a**); and their pan- (**b**) and core-genome (**c**) size evolution. The color scale next to the heatmap represents pairwise ANI values. The boxes in the boxplots represent the interquartile range of each group’s distribution of values; the lines inside the boxes represent the median values; the whiskers denote the lowest and highest values within 1.5 times the interquartile range of each group; the dots above/below the upper/lower line are the outliers.

The chromosome size of these genomes ranged from 2.83 to 3.27 Mb. The average nucleotide identity (ANI) of the 27 isolates was compared with the genome of *L. paracasei* Zhang, showing >98% sequence similarity (Fig. 1a), confirming their species-level taxonomy. The results of genome annotation identified 2652–3230 coding sequences (CDSs) per *L. paracasei* isolate. Their pan-genome consisted of 8250 gene families (Fig. 1b); and the size of the core-genome decreased with the increase in the isolate number (Fig. 1c).

### Adenine methylation is highly variable among *L. paracasei* isolates and is skewed toward carbohydrate metabolism-related genes

To understand the physiological roles of DNA methylation in *L. paracasei*, we first determined their genome-wide methylome profiles. To analyze the contribution of individual 6mA methyl groups, SMRT sequencing was applied to provide the kinetic information of the DNA polymerase, possible modification types and sequence motifs.

**Fig. 2:**
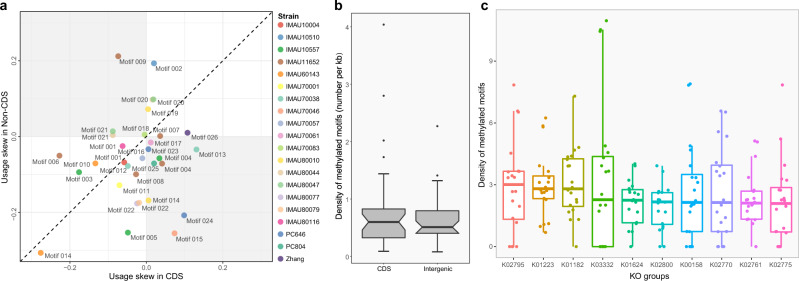
Motif usage bias analysis and distribution of methylation sites in *Lacticaseibacillus paracasei* isolates. Motif usage bias revealed by the Markov chain approach based on the analysis of 26 motifs identified in this study. The data of each strain/isolate are represented by a data dot of different color, and each motif is assigned to a specific motif number. The distance between a data dot and its projection on the diagonal line measures the extent of motif usage biases between coding sequences (CDSs) and intergenic regions. Data dots below and above the diagonal line represent skewing toward CDSs and the intergenic regions, respectively (**a**). Notched boxplots of the average frequency of 6mA methylation of the 26 motifs in the CDSs and the intergenic regions (**b**); distribution of methylation sites across CDSs of the top 10 Kyoto Encyclopedia of Genes and Genomes (KEGG) Orthology (KOs) of 20 *Lacticaseibacillus paracasei* isolates (**c**). The *x*- and *y*-axes represent the KO groups and methylation density, respectively. *X*-axis is sorted according to the median methylation density of the KO group. KO groups: K02795, mannose PTS system EIIC component; K01223, 6-phospho-beta-glucosidase; K01182, oligo-1,6-glucosidase; K03332, fructan beta-fructosidase; K01624, fructose-bisphosphate aldolase, class II; K02800, mannitol PTS system EIICBA or EIICB component; K00158, pyruvate oxidase; K02770, fructose PTS system EIIBC or EIIC component; K02761, cellobiose PTS system EIIC component; K02775, galactitol-PTS system EIIC component. The length of the box represents the range between the 25th and 75th percentile; the lines inside the boxes represent the median values; the whiskers denote the lowest and highest values within 1.5 times the interquartile range of each group; the dots above/below the upper/lower line are the outliers.

The isolates of *L. paracasei* contained greatly diverse and individualized 6mA methylated bases and motifs, ranging from 375 to 28,132 per isolate, and 78% of the 6mA sites were found in the identified motifs (Table 1). Among the 28 investigated isolates (including *L. paracasei* Zhang), 20 were found to possess methylation motifs, and a total of 26 different methylated motifs were identified. Many of the isolates contained unique motif sequence(s) (Table 1). Putative methylase genes present in the 28 isolates were annotated by REBASE, and, in most cases, the methylome signatures correlated well with the genome methyltransferase detected in the respective genomes (Supplementary Table S1). A Markov chain approach analysis revealed that the range of motif usage bias in the CDSs and the intergenic regions deviated from −0.518 to 0.212; and 20/32 methylated motifs were more often found in the CDSs compared with the intergenic regions (Fig. 2a). The average frequency of 6mA methylated motifs was non-significantly higher within the CDSs than the intergenic regions (0.95 versus 0.76 per kb; Fig. 2b).

**Table 1:** Information of detected methylated motifs and bases in different isolates

Isolate	Motif ID^a^	Motif sequence^b^	Number of methylated motifs	Total number of motifs	Proportion of methylated motifs (%)	Number of methylated bases^c^
IMAU10004	motif_001	CYYANNNNNNGTG/CACNNNNNNTRRG	1636	1652	99.03	1937
IMAU10510	motif_002	CGANNNNNNNTARC/GYTANNNNNNNTCG	1046	1066	98.12	1585
IMAU10557	motif_003	CTGCAG	1460	1476	98.92	13,707
	motif_004	GCATC/GATGC	9178	9338	98.29	
	motif_005	GCANNNNNNNTTAC/GTAANNNNNNNTGC	652	666	97.90	
IMAU11652	motif_006	CCATC/GATGG	6292	8100	77.68	28,132
	motif_007	CGGAT/ATCCG	5996	6127	97.86	
	motif_004	GCATC/GATGC	8911	8982	99.21	
	motif_008	TGGAG/CTCCA	2428	2442	99.43	
	motif_009	CGANNNNNNNTAYG/CRTANNNNNNNTCG	984	986	99.80	
IMAU60143	motif_010	GAGCC/GGCTC	2795	2797	99.93	3452
IMAU70001	motif_011	YAGGAG/CTCCTR	956	1018	93.91	1559
IMAU70038	motif_012	GCAAAG/CTTTGC	2516	2516	100.00	4423
	motif_013	GARANNNNNNGTG/CACNNNNNNTYTC	1358	1358	100.00	
IMAU70046	motif_014	ACCNNNNNNGTC/GACNNNNNNGGT	2251	2256	99.78	3444
	motif_015	CRTANNNNNNCGT/ACGNNNNNNTAYG	619	624	99.20	
IMAU70057	motif_016	CGANNNNNNNTTGY/RCAANNNNNNNTCG	1999	2012	99.35	2430
IMAU70061	motif_017	GCCAT/ATGGC	11,353	11,419	99.42	12,368
IMAU70083	motif_018	GYTANNNNNNNTTGY/RCAANNNNNNNTARC	953	956	99.69	2716
IMAU80010	motif_019	ACCNNNNNCCT/AGGNNNNNGGT	1924	1966	97.86	10,074
	motif_014	ACCNNNNNNRTC/GAYNNNNNNGGT	6575	7382	89.07	
IMAU80044	motif_020	AAGGAG/CTCCTT	1100	1142	96.32	3629
	motif_021	CCANNNNNNNTYTC/GARANNNNNNNTGG	1729	1750	98.80	
IMAU80047	motif_020	AAGGAG/CTCCTT	1093	1110	98.47	4706
	motif_021	CCANNNNNNNTYTC/GARANNNNNNNTGG	1727	1730	99.83	
IMAU80077	motif_022	CCANNNNNNNTANNG/CNNTANNNNNNNTGG	2716	2726	99.63	3096
IMAU80079	motif_022	CCANNNNNNNTANNG/CNNTANNNNNNNTGG	2693	2696	99.89	3535
IMAU80116	motif_001	CYYANNNNNNGTG/CACNNNNNNTRRG	1741	1808	96.29	2357
PC646	motif_023	GCANNNNNNNTGC	2216	2218	99.91	3746
	motif_024	GACNNNNNRTAT/ATAYNNNNNGTC	961	962	99.90	
PC804	motif_025	GCAAAT/ATTTGC	2722	2825	96.35	3857
Zhang	motif_026	ACRCAG/CTGYGT	1861	1908	97.54	2621

^a^Each motif ID represents one specific motif sequence.

^b^Palindromic motifs are presented in the format of “forward/reverse”.

^c^Eight isolates (IMAU10043, IMAU10685, IMAU30101, IMAU70017, IMAU70018, PC724, IMAU70027, and IMAU80048) have no detected motifs, and their methylated bases ranged from 375 to 1312. Degenerate bases: N = A, C, G, T; R = A, G; Y = C, T.

When mapping the methylated motifs to the annotated genomes of the *L. paracasei* isolates, 18 out of 20 isolates exhibited a skewed distribution of methylation motifs toward CDS of genes in the Clusters of Orthologous Groups of proteins (COG) functional category of carbohydrate transport and metabolism [G] and translation, ribosomal structure and biogenesis [J]. These two categories are more skewed than other COG categories. The COG functional category [J] contains genes that play important roles in bacterial methylation^6^, while the COG functional category [G] is related to carbohydrate metabolism. The COG functional category [G] genes of the 20 *L. paracasei* isolates with methylated motifs were inferred and mapped to 15 carbohydrate metabolism-related pathways (Supplementary Fig. S1). The frequency distribution of methylation motifs in these genes varied greatly between isolates, and the most methylated genes encoded 6-phospho-beta-glucosidase, oligo-1,6-glucosidase, fructan beta-fructosidase, fructose-bisphosphate aldolase, pyruvate oxidase, and some components of the mannose, fructose, cellobiose and galactitol phosphoenolpyruvate-dependent phosphotransferase systems (PTSs; Supplementary Fig. S2 and Fig. 2c). The skewed distribution pattern of methylated motifs among these genes is suggestive of an epigenetic level of carbohydrate metabolism regulation, accounting for the versatile growth behavior of *L. paracasei*.

### Adenine methylation regulated the expression of carbohydrate metabolism-coding genes at the transcriptomic and proteomic levels

Annotation using REBASE revealed two methyltransferases in *L. paracasei* Zhang, and these two methyltransferases are located in the bacteriophage exclusion system, which is an eight-gene cassette in the genome of *L. paracasei* Zhang (Supplementary Fig. S3). Eight of the 27 other investigated *L. paracasei* isolates in this study were found to possess an identical (IMAU30101 and IMAU10043) or a partial bacteriophage exclusion system with at least one missing gene (IMAU70083, IMAU70038, IMAU60143, IMAU70061, IMAU70017, IMAU70001; Supplementary Fig. S3). However, 6mA methylation in *L. paracasei* Zhang was obliterated only by inactivating its *pglX* gene but not the second methyltransferase, suggesting that 6mA methylation in *L. paracasei* Zhang is solely responsible by the *pglX* gene and that the second methyltransferase gene in this strain plays a regulatory role in the adenine methylation process^11, 17^.

Owing to the well-characterized physiological properties of *L. paracasei* Zhang^18^ and the availability of the *pglX* mutant, it was selected from the isolate set as a representative strain for further probing the potential role of 6mA methylation in carbohydrate metabolism. Although not drastic, an obvious growth difference was observed between the wild-type *L. paracasei* Zhang and the *pglX* mutant when they were grown in a chemically defined medium (CDM; Supplementary Fig. S4). The *pglX* mutant grew faster in the log phase (4–14 h) compared with the wild type, reflected by the higher OD values and lower pH values of *pglX* mutant during the entire period of this growth stage. We then profiled the transcriptomes and proteomes of the two strains after growing them in CDM for 12 h (late log phase) to decipher the molecular mechanism of the different growth behavior.

**Fig. 3:**
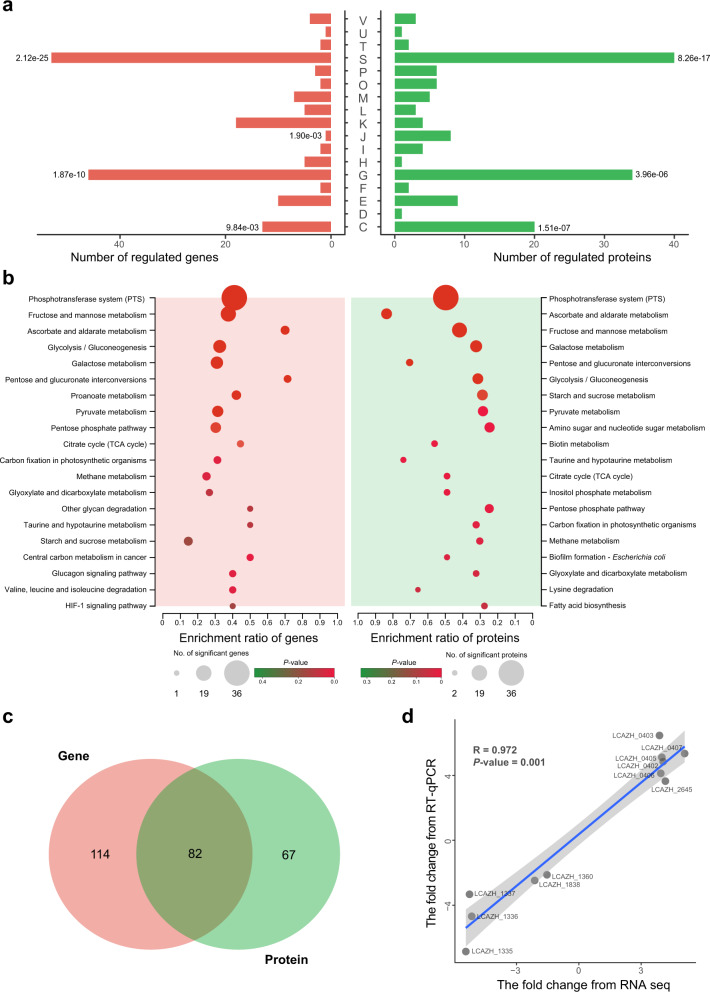
Analysis of differentially expressed genes (DEGs) and proteins (DEPs) across functional categories and pathways. Distribution and enrichment analysis of DEGs and DEPs (left and right panels, respectively) across different Cluster of Orthologous Group of proteins (COG) functional categories. The DEGs and DEPs were identified by transcriptomics and proteomics, respectively. Significant enrichment is indicated by a single asterisk (the *P* values less than 0.05 are labeled; two-sided Fisher’s exact test). All exact *P* values are provided in the data source file (**a**). COG functional categories: [C], Energy production and conversion; [D], Cell cycle control, cell division, chromosome partitioning; [E], Amino acid transport and metabolism; [F], Nucleotide transport and metabolism; [G], Carbohydrate transport and metabolism; [H], Coenzyme transport and metabolism; [I], Lipid transport and metabolism; [J], Translation, ribosomal structure and biogenesis; [K], Transcription; [L], Replication, recombination and repair; [M], Cell wall/membrane/envelope biogenesis; [O], Posttranslational modification, protein turnover, chaperones; [P], Inorganic ion transport and metabolism; [S], Function unknown; [T], Signal transduction mechanisms; [U], Intracellular secretion, trafficking, and vesicular transport; [V], Defense mechanisms. Kyoto Encyclopedia of Genes and Genomes (KEGG) pathway enrichment analysis of DEGs and DEPs, shown in the left and the right panels, respectively. Statistical difference was tested by two-sided Fisher’s exact test; and the color scale represents the *P* values. *P* values with correction by the Benjamini–Hochberg procedure are shown in the data source file (**b**). Venn diagram showing common differentially regulated genes between transcriptomics and proteomics (**c**). Pearson correlation analysis of data generated by real-time quantitative polymerase chain reaction (RT-qPCR) and RNA-seq. *X*- and *y*-axes show the data of fold-change found by RNA-seq and RT-qPCR, respectively. The gray-shaded area represents the error band of Standard Deviation (SD) (**d**).

At the late log phase, a total of 196 differentially expressed genes (DEGs) were identified, including 164 upregulated genes and 32 downregulated genes (Supplementary Table S2). The results of COG and Kyoto Encyclopedia of Genes and Genomes (KEGG) gene annotation and enrichment analysis showed that the DEGs were mostly related to carbohydrate transport and metabolism (left panel of Fig. 3a, b). At the proteomic level, a total of 149 DEPs were identified, including 126 significantly increased and 23 significantly decreased proteins (Supplementary Data 1). The results of transcriptomic and proteomic analyses were largely consistent, revealing obvious enrichment in DEPs involved in carbohydrate transport and metabolism (right panel of Fig. 3a, b). A total of 82 DEGs/DEPs were shared by the two data sets, including: 78 significantly increased genes/proteins and two significantly decreased gene/protein (Fig. 3c, Supplementary Table S2 and Supplementary Data 1). Most likely, the differential expression of these carbohydrate metabolism-related genes/proteins is responsible for the phenotypic growth variation between *L. paracasei* Zhang and the *pglX* mutant.

For validation, 11 DEGs from the transcriptomic data were randomly selected for real-time quantitative PCR analysis. Data generated by real-time quantitative PCRs and transcriptomic analysis showed good congruence (R = 0.972; *p* = 0.001; Fig. 3d), suggesting high reliability of the transcriptomic analysis. Notably, 6 of the 11 selected genes also showed consistent proteomic-level differential expression trends.

**Fig. 4:**
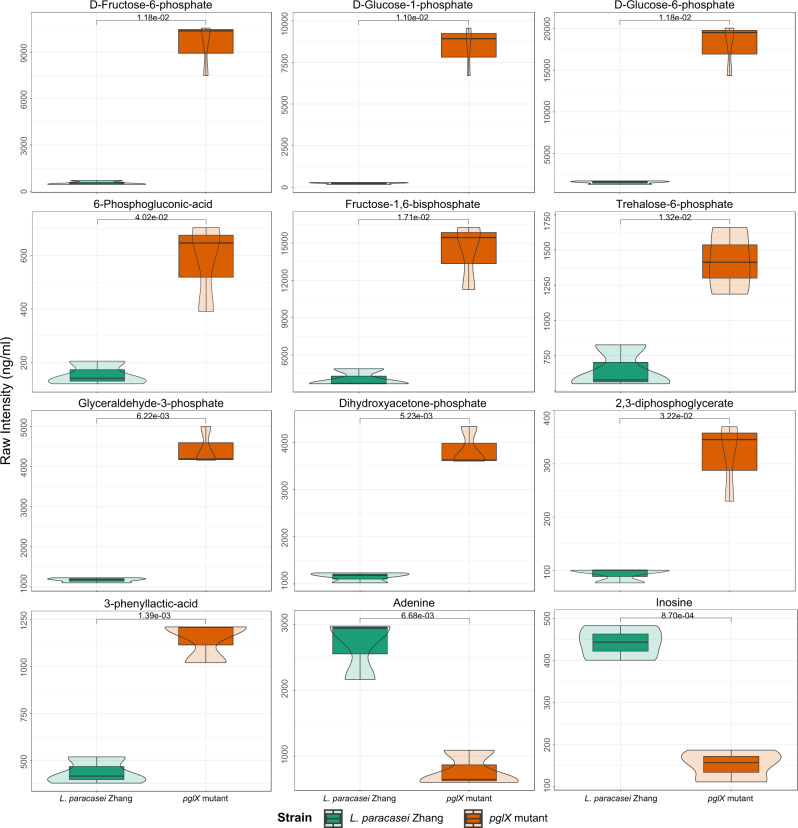
The signal intensity of 12 significant differential metabolites identified between *Lacticaseibacillusparacasei* wild type (reference condition, *n* = 3) and *pglX* mutant (test condition, *n* = 3). In each violin graph, the *x*-axis indicates the bacterial strain; and the *y*-axis indicates the raw signal intensity of detected metabolites. The width of the violin plot is proportional to the estimated density of the observed data. The bottom and top of the box show the 25th and 75th percentile; and the horizontal line in the box indicates the median value. Statistical difference was evaluated by *t*-test (two-sided). The *P* values less than 0.05 are labeled, and all exact *P* values are provided in the data source file.

Adenine methylation-mediated regulation of carbohydrate metabolism in *L. paracasei* was also confirmed by metabolomic analysis, which revealed significant increases in the levels of various carbohydrate metabolites in the late log phase culture of *pglX* mutant, including D-fructose-6-phosphate, D-glucose-1-phosphate, D-glucose-6-phosphate, 6-phosphogluconic-acid, fructose-1,6-bisphosphate, trehalose-6-phosphate, glyceraldehyde-3-phosphate, dihydroxyacetone-phosphate, 2,3-diphosphoglycerate, and 3-phenyllactic-acid (Fig. 4). The metabolomic results serve as a strong support for the observations of our transcriptomics and proteomics analyses.

### Adenine methylation affects the spatial genome organization of carbohydrate metabolism-related genes

It has been reported that spatial genome organization is important in regulating gene/protein expression^19^. Thus, mapping the chromatin topology of *L. paracasei* Zhang *pglX* mutant and its wild type would provide novel insights into the cellular and molecular regulation of activities of lactobacilli from a fresh perspective. Comparative Hi-C analysis of *L. paracasei* Zhang *pglX* mutant and its wild type from log-phase cultures was therefore performed to uncover the link between 6mA methylation and chromosome topology. A total of 95,586,301 and 102,778,323 clean read-pairs were produced for *L. paracasei* Zhang *pglX* mutant and its wild type, respectively.

**Fig. 5:**
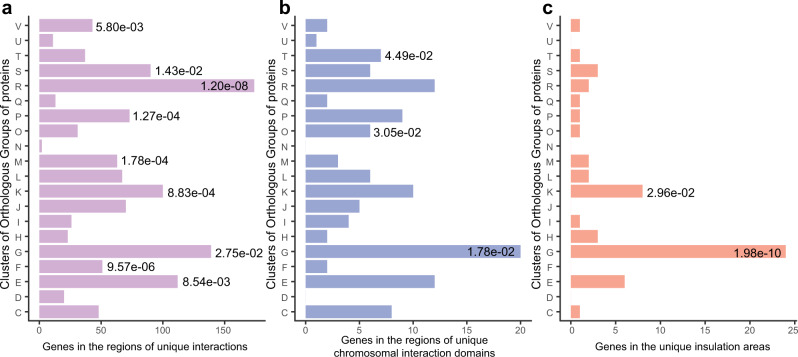
Distribution and enrichment analysis of Cluster of Orthologous Group of proteins (COG) of differentially expressed genes. *X*-axis indicates the number of genes in the regions of unique interactions (**a**), unique chromosomal interaction domains (**b**), and unique insulation areas (**c**) were analyzed. Significant gene enrichment in a specific functional category is indicated by a single asterisk (the *P* values less than 0.05 are labeled; two-sided Fisher’s exact test). All *P* values are provided in the data source file. COG functional categories: [C], Energy production and conversion; [D], Cell cycle control, cell division, chromosome partitioning; [E], Amino acid transport and metabolism; [F], Nucleotide transport and metabolism; [G], Carbohydrate transport and metabolism; [H], Coenzyme transport and metabolism; [I], Lipid transport and metabolism; [J], Translation, ribosomal structure and biogenesis; [K], Transcription; [L], Replication, recombination and repair; [M], Cell wall/membrane/envelope biogenesis; [N], Cell motility; [O], Posttranslational modification, protein turnover, chaperones; [P], Inorganic ion transport and metabolism; [Q], Secondary metabolites biosynthesis, transport and catabolism; [R], General function prediction only; [S], Function unknown; [T], Signal transduction mechanisms; [U], Intracellular secretion, trafficking, and vesicular transport; [V], Defense mechanisms.

In both the genome-wide contact heatmaps of *L. paracasei* Zhang *pglX* mutant and its wild type (Supplementary Fig. S5a, b), a single intense diagonal was observed, suggesting a smaller distance with a higher contact probability between adjacent chromosome regions. In general, 1215 interactions, 16 chromosomal interaction domains (CIDs), and 14 insulation areas were unique in the *pglX* mutant, representing 1327, 132, and 62 coding genes, respectively. Interestingly, genes involved in the COG functional category of carbohydrate transport and metabolism [G] were enriched in all three types of chromosomal interacting regions (Fig. 5), and a number of these genes showed altered expression in the transcriptomics or proteomics analyses (Supplementary Data 2). Our data revealed the potential effect of 6mA methylation on the spatial genome organization and the gene expression in the affected genomic regions.

### Adenine methylation of transcription factor binding sites (TFBSs) upstream of carbohydrate metabolism-related genes

To further elucidate how methylation regulates carbohydrate metabolism, we searched the *L. paracasei* genomes for TFBSs and comparatively analyzed their methylation profiles. Our search returned 330–540 TFBS-containing upstream regions of CDSs across the 28 *L. paracasei* genomes, and most of the identified genomic regions carried conserved motifs for binding to transcriptional regulators like *CcpA*, *RbsR*, and *GalR* (Supplementary Data 3). Then, to identify conserved TFBSs present in specific genes, we performed another homology search using sequences of upstream TFBSs together with their downstream CDSs, which returned 82 putative functional or hypothetical proteins, and 78 of which were associated with non-methylated sites (Supplementary Data 4). Again, many of these downstream CDSs encoded carbohydrate metabolism-related genes, including N-acetylglucosamine-6-phosphate deacetylase, alpha-glucosidase, glycoside hydrolase family 65 protein, aldose 1-epimerase family protein, PTS sugar transporter subunit IIA, PTS sugar transporter subunit IIB, and some predicted transcriptional regulators (Supplementary Data 4).

We next mapped the TFBSs to the upstream regions of the DEGs identified in the *pglX* mutant and compared them against the methylation profile of *L. paracasei* Zhang. Notably, in the *pglX* mutant, the upstream region of the pyruvate dehydrogenase (PDH) complex gene cluster, encoding the PDH (acetyl-transferring) E1 component subunit alpha (LCAZH_1299, *pdhA*), the PDH E1 component beta subunit (LCAZH_1300, *pdhB*), the dihydrolipoamide acetyltransferase (LCAZH_1301, *pdhC*), and the dihydrolipoyl dehydrogenase (LCAZH_1302, *pdhD*), contained a non-methylated motif that was highly similar to the *CcpA* binding site present in *L. paracasei*, according to the information retrieved from the RegPrecise database. Such observation is suggestive of the role of adenine methylation in regulating pyruvate metabolism in the *pglX* mutant.

## Discussion

Our results of whole-genome sequencing and methylomics analyses showed that adenine methylation is variable among *L. paracasei* isolates and is skewed toward carbohydrate metabolism-related genes, particularly genes coding for key enzymes and components of PTSs. The central role of PTSs is the nutrient acquisition, particularly in the processes of carbohydrate transportation and phosphorylation^16^. The skewed distribution pattern of methylated motifs among this group of functional genes drove us to hypothesize that there was an epigenetic level of regulation of carbohydrate metabolism in *L. paracasei*, accounting for its growth versatility under nutrient-limiting conditions.

Our previous study found that the wild type and its *pglX* mutant (a *pglX* gene-inactivated strain) exhibited no significant difference in growth performance in de Mann Rogosa Sharpe medium, a nutrient-rich medium^11^. We then tested our hypothesis by comparing the transcriptome, proteome, and spatial genome organization of the wild-type *L. paracasei* Zhang and its mutant grown in a nutritional restrictive CDM. As a common food use bacterium, *L. paracasei* is also often subjected to nutrient-limiting or even deficit conditions during the food fermentation or production process. In fact, such environmental conditions can contribute to the flavor development of specific food products via altered bacterial growth and metabolic responses^13^. The *pglX* gene is responsible for 6mA methylation in *L. paracasei* Zhang; thus, the mutant lacks the ability to methylate adenine in the genome. Interestingly, the inactivation of *pglX* gene in *L. paracasei* affected mostly the gene expression of carbohydrate metabolism-related genes on the transcriptomic and proteomic levels. Metabolomics analysis detected more carbohydrate substrates of various types in the late log phase culture of *pglX* mutant than that of the wild type. Moreover, Hi-C analysis revealed the presence of carbohydrate metabolism-related genes in the unique interaction, CID, and insulation regions of the *pglX* mutant (Supplementary Data 2). These findings together suggested that 6mA methylation is involved in the regulation of carbohydrate metabolism in *L. paracasei* at the transcriptomics, proteomics, and metabolomics levels, and such regulation could be exerted via modulating the spatial genome organization. Spatial positions of regulatory sequences and proteins are important for regulating gene expression^20^, and the methylation of a local motif site does not only affect the expression of a proximal gene but also a distal one as a result of the tridimensional conformation of the chromosome^20^.

Notably, most of the investigated *L. paracasei* isolates have unique 6mA methylation patterns in their carbohydrate metabolic genes, which might be one of the mechanisms for their differential gene regulation of carbohydrate metabolism. Intragenic methylation of hemi-methylation clusters has been proposed as a regulatory mechanism of gene expression in bacteria like *Escherichia coli* via elevating the melting temperature^21^. A recent study by Hua et al. (2022) has provided further evidence of intragenic transcriptional regulation by direct binding of transcriptional factors to the coding regions to modulate transcription of the bound or adjacent genes^22^. García-Pastor et al. (2019) reported the bistable expression of the *Salmonella enterica std* fimbrial operon through a competitive regulatory control between DNA adenine methylation and formation of the StdE-StdF-HdfR activator loop upstream of the *std* promoter^23^. Thus, transcription is regulated via complicated mechanisms involving both upstream and/or the coding region of a gene/operon, and such control could be directed via modifying specific methylation sites and/or TFBSs. The current comparative analysis of TFBSs across the genomes of *L. paracasei* identified several conserved TFBSs with non-methylated sites at the upstream regions of carbohydrate metabolism-related genes, implicating the existence of putative interactive gene regulatory mechanisms mediated by a combined action of transcriptional factor binding and methylation, although the observation of colocalization of the methylation sites and differentially expressed carbohydrate metabolic genes alone does not allow disentangling a direct from a pleiotropic effect. The overlapping between non-methylated sites and known TFBSs would be more direct evidence for the regulation of gene expression. Nevertheless, the possession of both TFBSs and methylation sites in these regions suggested that these features are likely playing a role in governing the cellular and metabolic responses and confining the species-/strain-specific carbohydrate metabolic capacity of *L. paracasei*.

As expected, genes encoding key enzymes involved in the carbohydrate metabolism were identified, and some of which were significantly upregulated in the *pglX* mutant compared with the wild type, including class II fructose-bisphosphate aldolase (LCAZH_0191, LCAZH_0381, and LCAZH_2698), triose-phosphate isomerase (LCAZH_2697), aldose 1-epimerase (LCAZH_1782, LCAZH_2563), acetate kinase (LCAZH_0188), xylulokinase (LCAZH_0190), PDH (acetyl-transferring) E1 component subunit alpha (LCAZH_1299, *pdhA*), PDH E1 component beta subunit (LCAZH_1300, *pdhB*), dihydrolipoamide acetyltransferase (LCAZH_1301, *pdhC*), dihydrolipoyl dehydrogenase (LCAZH_1301, *pdhD*), and L-lactate dehydrogenase (LCAZH_0554). Class II fructose-bisphosphate aldolase, triose-phosphate isomerase, and aldose 1-epimerase are enzymes of the glycolytic pathway, while acetate kinase, xylulokinase, and PDH participate in pyruvate metabolism. Moreover, an increase in the activity of the PDH complex would enhance the conversion of glucose to pyruvate by L-lactate dehydrogenase to produce lactic acid^24^. These proteins are anticipated to be highly expressed in actively proliferating bacteria to ensure enough energy production for supporting growth. Moreover, the enhanced expression of carbohydrate metabolism-related genes and proteins in the *pglX* mutant is also accompanied by obvious increases in a multitude of carbohydrate metabolites compared with the wild type. Intriguingly, a *CcpA* motif from *L. paracasei* was discovered in the upstream region of the PDH complex gene cluster in the *pglX* mutant with no methylated sites. Regulatory proteins bind more favorably to non-methylated DNAs with the highest affinity, enhancing their interactions and effectiveness in modulating the expression of downstream genes and metabolic pathways^25^. We thus speculate that the upregulation of the PDH complex was mediated by the *CcpA* motif with non-methylated sites.

The omics data set also showed the upregulation of several genes in the vitamin C (L-ascorbate) metabolic pathway, including L-ribulose-5-phosphate 4-epimerase (LCAZH_2733 and LCAZH_2735), DeoR/GlpR transcriptional regulator (LCAZH_2736), 3-keto-L-gulonate-6-phosphate decarboxylase (LCAZH_2737), *Ula*A (LCAZH_0192, LCAZH_0379 and LCAZH_2739), *Ula*B (LCAZH_0378 and LCAZH_2738), and *Ula*C (LCAZH_0377 and LCAZH_2740) in the *pglX* mutant compared with the wild type. Under anaerobic conditions, vitamin C could serve as a sole carbon source for supporting bacterial growth. In *E. coli*, the catabolic pathway of vitamin C is encoded by an operon containing six genes, namely *ulaABCDEF*, encoding a transporter (*Ula*A), an IIB-like enzyme (*Ula*B), and an IIA-like enzyme (*Ula*C), which are necessary for bacterial uptake and phosphorylating vitamin C into L-ascorbate 6-phosphate^26^. Inactivating the genes encoding enzyme IIA and enzyme IIB of vitamin C-specific PTSs in *Streptococcus mutans* would extend the growth lag phase and decrease the growth yield in vitamin C-containing medium^27^. The differential regulation of these genes suggested that 6mA methylation can also regulate the metabolic pathway of vitamin C utilization.

Unlike PTSs of other families, the mannose PTS has an IID protein. Bacterial mannose PTSs have been shown to have broad substrate specificity, such as mannose, glucose, and galactose^28^. Mannose PTSs in some lactobacilli have previously been characterized. Functionally, the mannose PTSs in *Latilactobacillus curvatus* have been proven to be glucose and mannose transporters, though no glucose-specific PTS activity was found^29^. Similarly, a 2-deoxy-D-glucose-resistant mutant of *L. paracasei* was found to be impaired in the main glucose transport mechanism^30^, and thus the mannose PTSs in the *pglX* mutant are likely functioned via a strong alternative catabolite repression mechanism by glucose and mannose of the lactose and ribose assimilation genes^30^. Our data showed an upregulation of mannose PTSs-coding genes in the *pglX* mutant, including fructose/mannose PTS IIA component (LCAZH_0402), PTS sugar transporter subunit IIB (LCAZH_0403), PTS sugar transporter subunit IIC (LCAZH_0404), PTS system mannose/fructose/sorbose family transporter subunit IID (LCAZH_0405), and PTS sugar transporter (LCAZH_0406). In addition, it is interesting to note that there was an increase in the expression of the N-acetyl-galactosamine (Aga)-series components of the mannose PTSs in the *pglX* mutant, namely PTS fructose transporter subunit IIA (LCAZH_0402 and LCAZH_2662), PTS system mannose/fructose/sorbose family transporter subunit IID (LCAZH_2663), PTS sugar transporter subunit IIC (LCAZH_2664), and PTS sugar transporter subunit IIB (LCAZH_2665). In *E. coli*, Aga-PTSs are responsible for N-acetyl-galactosamine and galactosamine utilization^31^. These amino sugars are particularly required for bacterial cell wall synthesis. Therefore, the increase in uptake efficiency in these sugars by regulating Aga-PTSs in *L. paracasei* is likely a protective mechanism activated in the late log phase for cell maintenance.

Another 6mA methylation-regulated PTS-related gene set at both transcriptomic and proteomic levels was the galactitol family, including PTS galactitol transporter subunit IIC (LCAZH_2647), PTS galactitol transporter subunit IIB (LCAZH_2648), and PTS sugar transporter subunit IIA (LCAZH_2649). Bacterial galactitol-PTSs are associated with D-arabitol utilization. Although this gene cluster has been annotated as CDSs for galactitol fermentation, most available evidence supported that this subset of gene is related to arabitol metabolism. For example, genes encoding galactitol-PTSs have been found to be highly activated during the growth of *Bacillus methanolicus* utilizing arabitol as the sole carbon source^32^. The role of galactitol-PTS transporter in *L. paracasei* and the exact reason for its differential regulation by 6mA methylation would require further investigation.

The whole-genome sequencing and methylomics analyses revealed great variation in 6mA methylation pattern among *L. paracasei* strains, and such variation between strains could potentially account for the strain-specificity and versatility in carbohydrate metabolism of this species. Data from further multi-omics and Hi-C analyses of the *L. paracasei* wild type and its *pglX* mutant consistently supported that 6mA methylation could be a regulatory mechanism for its carbohydrate metabolism. Our study provides new insights into the role of 6mA methylation in *L. paracasei*, particularly with prior knowledge that carbohydrate metabolism affects both the growth and survival of *L. paracasei* under various environmental conditions. Further study to understand the role of epigenomic regulation of the growth and activity of *L. paracasei* would be of interest in improving industrial production using this species.

## Methods

### Bacterial strains and cultivation

Twenty-eight *L. paracasei* isolates (including *L. paracasei* Zhang) and a *pglX* gene-inactivated strain of *L. paracasei* Zhang were obtained from the Key Laboratory of Dairy Biotechnology and Engineering, Ministry of Education, at the Inner Mongolia Agricultural University of China. For strain activation, the bacteria were cultivated in standard de Mann Rogosa Sharpe (MRS) broth (CM0359; Oxoid, Ltd., Basingstoke, UK). For RNA-seq analysis, proteomics analysis, Hi-C, and metabolomics analysis, the bacteria were cultivated in a CDM (Supplementary Table S3). The CDM was a minimal medium developed for investigating the growth and metabolism of *L. paracasei*^33^. The growth of *L. paracasei* Zhang and the *pglX* mutant in CDM were measured by changes in pH and optical density at 600 nm (OD_600_).

### Genomics and methylomics analyses by Illumina and SMRT sequencing

Genomic DNA was isolated by the Wizard Genomic DNA Purification Kit (Promega, Madison, WI, USA). The integrity of DNA was examined by 0.6% agarose gel and 1.2% Lonza FlashGel electrophoresis. For SMRT sequencing, libraries with an insert size of 10 kb were constructed using the PacBio SMRTbell TM Template Kit. The quality of the libraries was evaluated on a Qubit® 2.0 Fluorometer (Thermo Fisher Scientific, Waltham, MA, USA), and the insert fragment size was determined by an Agilent 2100 Bioanalyzer (Agilent Technologies, Inc., Santa Clara, CA, USA). For Illumina sequencing, libraries were prepared using the NEBNext® Ultra™ DNA Library Prep Kit (New England Biolabs, Inc., Ipswich, MA, USA). The DNA samples were first fragmented by sonication to a size of around 350 bp. Then, the DNA fragments were end-polished, A-tailed, and ligated with the full-length adaptor by PCR amplification. The PCR products were purified with AMPure XP system, and the quality and size distribution of libraries were evaluated by an Agilent 2100 Bioanalyzer. Sequencing was performed on a PacBio Sequel platform (Pacific Biosciences of California, Inc., Menlo Park, CA, USA) and an Illumina NovaSeq 6000 (Illumina, Inc., San Diego, CA, USA), respectively.

De novo assemblies were realized by a standard hierarchical genome assembly process using only PacBio sequencing data from a single, long-insert library; and the consensus was called across reads after assembly polishing. Effective data of each sample after quality control were used to assemble the genome of reads by SMRT link v5.1.0 software, and the preliminary assembly results could reflect the crude genome quality of samples. Then, Arrow software (Pacific Biosciences of California, Inc., Menlo Park, CA, USA) was used to optimize the assembly results and correct areas with assembly errors by comparing the original data of the initial assembly sequence against data generated by the Illumina platform^34,35^. The chromosomal and plasmid sequences were identified, and chromosomal sequences were assembled into a circular genome. To identify base modifications and methyltransferase motifs, the protocols for modification and motif analysis in SMRT Link software were used with the identification quality score ≥20^36^. Methylation sites generated by the protocol were mapped to the genomes. Methyltransferases were identified by REBASE using BLASTP with identity >50%, e value <1e–10, and bit score >50^37^.

Gene prediction was realized in Prokka (version 1.13) with the argument of kingdom Bacteria^38^. Functional annotation of coding sequences (CDSs) was conducted by using the databases of Rapid Annotation Subsystem Technology (RAST) 2.0^39^, KEGG^40^, and COG^41^. The ANI was calculated by a standalone java ANI calculator^42^. The skewness of CDS and COG distribution was evaluated with a Markov model that considered motif composition^36^. Motif-based sequence analysis was performed by the MEME suite (v5.0.5)^43^. First, the upstream regions with a length of 50–300 bp of *L. paracasei* genes were extracted using a python script, intergenic_regions.py^44^. A *Lactobacillaceae*-specific TFBS catalog was built by using the sites2meme script of MEME suite based on motif sequences, which included 82 transcription factor regulons of 15 *Lactobacillaceae* strains. Then, the FIMO tool included in the MEME suite was used to scan upstream regions of *L. paracasei* genes for the occurrence of putative TFBSs with the *q* value (adjusted *P* value) threshold of 0.05^45^. The motif sequence logo was constructed by WebLogo3^46^.

### RNA-seq analysis

Triplicate parallel cultures of wild-type *L. paracasei* Zhang (reference condition) and *pglX* mutant (test condition) were grown in the CDM to late log phase, and bacterial cells were harvested. Total RNA was extracted using the Trizol reagent (Invitrogen Corporation, Carlsbad, CA, USA) following the manufacturer’s instructions. The RNA library was constructed from 2 μg of total RNA using the TruSeq^TM^ RNA Sample Preparation Kit (Illumina Inc., San Diego, CA, USA). Briefly, rRNA was removed from the total RNA by a Ribo-Zero Magnetic Kit (Epicenter Biotechnologies, Madison, WI, USA), and the mRNAs were randomly fragmented into lengths of about 200 nucleotides. Double-stranded cDNA was synthesized by reverse transcription using random hexamer primers (Illumina Inc., San Diego, CA, USA) and a SuperScript Double-stranded cDNA Synthesis Kit (Invitrogen Corporation, Carlsbad, CA, USA). Phusion DNA polymerase (New England Biolabs, Inc., Ipswich, MA, USA) was used for PCR amplification by a total of 15 cycles. After the library was quantified by the Turner BioSystems TBS-380 Mini-Fluorometer (in conjunction with Molecular Probes’ PicoGreen® dsDNA Quantitation Reagent), Illumina HiSeq X Ten was used for RNA-seq paired-end sequencing.

Clean reads were obtained by removing the adapter sequences, filtering low-quality sequences at the end of the reads, and removing reads with N ratio of 10%. The high-quality clean reads were mapped to the reference genome by using Bowtie2 (http://bowtie-bio.sourceforge.net/bowtie2/index.shtml). In addition, 10,000 raw reads were randomly selected from each sample and compared against the Rfam database (http://rfam.xfam.org/) using BLAST. The rRNA contamination rate in the samples was calculated based on the annotation results. DESeq2 software (http://bioconductor.org/packages/release/bioc/html/DESeq2.html) was used to identify DEGs between samples (with a cut-off false discovery rate [FDR] of ≤0.05 and 2.0-fold change).

Real-time quantitative PCRs were performed to validate the RNA sequencing results. The RNA of three biological replicates of the collected samples was extracted by using the RNAprep Pure Cell/Bacteria Kit (Tiangen Biotech Co., Ltd., Beijing, China). Then 500 ng of RNA was reverse transcribed into cDNA with a reverse transcription kit (PrimeScript RT Reagent Kit with gDNA Eraser; Takara Biomedical Technology Co., Ltd., Beijing, China) according to the manufacturer’s instructions. Quantitative analysis was conducted via the qTOWER3G Touch Real-Time PCR System (Analytik Jena AG, Jena, Germany). The reaction was performed in a 20 μL system, containing 1 µL of cDNA template, 10 µL of SYBR Premix Ex TaqII (Takara Biomedical Technology Co., Ltd., Beijing, China), 0.8 µL of each primer, and 7.4 µL of ddH_2_O. The PCR conditions were as follows: initial denaturation at 95 °C for 30 s, 40 cycles of denaturation at 95 °C for 5 s, primer annealing, and DNA extension at 60 °C for 30 s. The housekeeping gene, glyceraldehyde phosphate dehydrogenase, was used as the reference gene. Comparative threshold cycle method (2^−ΔΔCT^) was used to calculate the relative gene expression level^47^. The primers used are listed in Supplementary Data 5.

### Proteomics analysis

Three biological replicates of culture samples of wild-type *L. paracasei* Zhang (reference condition) and *pglX* mutant (test condition) grown to late log growth phase in CDM were prepared. For protein extraction, samples were dissolved in the extraction buffer (1% sodium deoxycholate, 200 mM dithiothreitol, 50 mM Tris-HCl) containing protease inhibitors. Protein concentrations were assayed by a Pierce bicinchoninic acid protein assay kit (Thermo Fisher Scientific, Waltham, MA, USA). After reduction, cysteine alkylation and digestion, samples were labeled with tandem mass tag reagent (TMT reagent; Thermo Fisher Scientific, Waltham, MA, USA) according to the manufacturer’s instructions. Pooled samples were separated by an ACQUITY UPLC BEH C18 column (1.7 µm, 2.1 mm × 150 mm; Waters, Milford, MA, USA). Proteomic analyses were performed on an Easy-nLC system coupled to a Q Exactive HF-X (Thermo Fisher Scientific, Waltham, MA, USA) for 60 min. The peptides were dissolved in mass spectrometric loading buffer and separated on the C18-reversed phase column (75 μm × 25 cm, Thermo Fisher Scientific, Waltham, MA, USA) for 120 min at a volume flow rate of 300 nL/min; the mobile phases consisted of aqueous solution A (2% acetonitrile with 0.1% formic acid) and B (80% acetonitrile with 0.1% formic acid). The peptides were eluted using the following gradient: 0–67 min, 6–23% B; 67–81 min, 23–29% B; 81–90 min, 29–38% B; 90–92 min, 38–48% B; 92–93 min, 48–100% B; 93–120 min, 100–0% B. The Q Exactive HF-X was run in the collection mode of data-dependent acquisition. The mass spectrometry (MS) spectra (m/z 350-1500) were obtained with primary MS resolution 120000. The automatic gain control (AGC) was targeted at 3e6, and the maximum fill time was 50 ms. The top 15 intense precursor ions were selected into collision cell for fragmentation by higher-energy collision dissociation. The MS/MS resolution was set at 45,000; the AGC target was 2e5; the maximum fill time was 120 ms; the fixed first mass was 110 m/z; the minimum AGC target was 1e4; the intensity threshold was 8.3e4; and the dynamic exclusion time was 30 s.

Raw data of LC-MS/MS spectra were analyzed by Proteome Discover^TM^ Software 2.4. The MS/MS search criteria were as follows: precursor mass tolerance of 20 ppm; fragment mass tolerance of 0.02 Da; trypsin as the enzyme with 2 missed cleavage allowed; carbamidomethyl (C), TMTpro (K), and TMTpro (N-terminus) as static modifications; and oxidation (M), acetyl (N-terminus), met-loss (N-terminus), and met-loss with acetyl (N-terminus) as dynamic modifications. The cut-off FDR of peptide identification was ≤0.01. For protein identification, each protein should match at least one unique peptide. Proteins displaying a *P* value of <0.05 by *t*-test were considered statistically significant. A 1.2-fold change was defined as the threshold for regulated proteins.

### Hi-C analysis

The wild-type *L. paracasei* Zhang (reference condition) and *pglX* mutant (test condition) were grown to the late log phase in a CDM. Cells were collected by centrifugation, washed at room temperature, and crosslinked with 3% formaldehyde for 30 min. The formaldehyde was quenched with 0.375 M glycine for 20 min at 4 °C. The fixed cells were collected and stored in a −80 °C freezer. For library construction, the fixed cells were suspended in 100 µL Tris-EDTA buffer with 2 µL of lysozyme (Ready-Lyse™ Lysozyme Solution; Epicenter Biotechnologies, Madison, WI, USA). After incubation for 20 min, sodium dodecyl sulfate was added to lyze cells for 10 min at 65 °C. The lysed cells were digested in the reaction mixture consisting of 300 µL water, 50 µL 10-fold NEB buffer 2.1 (New England Biolabs, Inc., Ipswich, MA, USA), and 100 U of *Sau*3AI. Restriction fragment ends were labeled with biotinylated cytosine nucleotides by biotin-14-dCTP (TriLINK Biotechnologies, San Diego, CA, USA). After blunt-end ligation, proteinase K was used for reversing cross-linking overnight. The DNA was purified using the QIAamp DNA Mini Kit (Qiagen GmbH, Hilden, Germany) and sheared to a length of ~400 bp. Point ligation junctions were pulled down using Dynabeads® MyOne™ Streptavidin C1 (Thermo Fisher Scientific, Waltham, MA, USA). The Hi-C library was prepared by NEBNext® Ultra™ II DNA library Prep Kit (New England Biolabs, Inc., Ipswich, MA, USA) and was submitted for sequencing on an Illumina HiSeq X Ten platform (Illumina Inc., San Diego, CA, USA).

To avoid any artificial bias, quality filtering was realized by Trimmomatic software version 0.38, and then the clean data were iteratively aligned to the reference genome^48^. Valid paired reads were binned into nonoverlapping genomic intervals to construct contact maps. After the statistics of valid contacts at a defined resolution, an observed interaction matrix was obtained and normalized with an iterative normalization method. The contacts at the resolution of 1 kb bins were imported to Fit-Hi-C software for calculating the cumulative probability *P* value and FDR (*q* value). Significant interactions were discriminated by: *p* and *q* values of less than 0.01, and contact count >2^49^. CIDs are contiguous regions with a high degree of self-association, which were identified by dividing the chromosome into windows with fixed length using an insulation score algorithm^50^. Differential insulation areas were obtained by using the sliding-window method^49^. According to the insulation score of bins, the Pearson correlation coefficient of each window between two samples was calculated^49^. Windows with Pearson coefficient >0.6 were merged, and the remaining bins in the genome were regarded as the unique insulation regions^49^. Interactions, and CIDs that occurred only under the reference condition (in wild type *L. paracasei* Zhang but not the *pglX* mutant) was considered to be unique to the test condition and vice versa.

### Targeted metabolomics analysis of metabolites involved in energy metabolism

Samples of wild-type *L. paracasei* Zhang (reference condition) and *pglX* mutant (test condition) prepared from cells grown to late log phase in CDM were separated by an ACQUITY UPLC BEH Amide column (1.7 µm, 2.1 × 100 mm; Waters, Milford, MA, USA). The solvent system consisted of water with 10 mM ammonium acetate and 0.3% ammonium hydroxide (A), and 90% acetonitrile/water (B). The gradient was as follows: 0–1.2 min, 95% B; 8 min, 70% B; 9–11 min, 50% B; 11.1–15 min, 95% B.

Linear ion trap and triple quadrupole scans were carried out on a QTRAP® 6500+ LC-MS/MS System coupled to an electrospray ionization (ESI) turbo ion-spray interface. It was operated in both positive and negative ion modes. The operation conditions for ESI source were as follows: ion source, ESI±; source temperature, 550 °C; ion-spray voltage, 5500 V (positive), −4500 V (negative); curtain gas, 35 psi. Metabolites in energy metabolism were analyzed using multiple reaction monitoring (MRM). Data acquisition was realized using Analyst 1.6.3 software (Sciex, Framingham, MA, USA). Multiquant 3.0.3 software (Sciex, Framingham, MA, USA) was used to quantify metabolites. Mass spectrometer parameters, such as the declustering potentials and collision energies for individual MRM transitions, were optimized. A specific set of MRM transitions were monitored for each period according to the metabolites eluted within this period. Metabolite identification was based on the MetWare online platform (http://www.metware.cn/). Differentially regulated metabolites in energy metabolism between samples were determined by variable importance in projection and fold change.

### Reporting summary

Further information on research design is available in the Nature Portfolio Reporting Summary linked to this article.

## Supplementary information

Supplementary Information

Description of Additional Supplementary Files

Supplementary Data 1

Supplementary Data 2

Supplementary Data 3

Supplementary Data 4

Supplementary Data 5

Reporting Summary

## Data Availability

The raw data of *L. paracasei* strains generated by Illumina and SMRT sequencing have been deposited in the National Center of Biological Information (NCBI) Sequence Read Archive (SRA; http://trace.ncbi.nlm.nih.gov/Traces/sra/sra.cgi) under the accession numbers: SRR16925174-SRR16925228. The genome sequence of *L. paracasei* Zhang was retrieved from the NCBI GenBank under the accession number CP001084.2. The MS proteomics data have been deposited to the ProteomeXchange Consortium via the PRIDE partner repository (http://www.ebi.ac.uk/pride; data set identifier, PXD026826). The transcriptomics data have been deposited in the NCBI SRA under the accession number PRJNA725355. The Hi-C data of *L. paracasei* Zhang and its mutant have been deposited in the NCBI SRA under the accession number SAMN23078205. Source Data are provided with this paper.

## Source data

Source Data
