# Supplementary material for: Roles of adenine methylation in the physiology of Lacticaseibacillus paracasei
Source: Nat Commun. 2023 May 6;14:2635. doi: 10.1038/s41467-023-38291-1 (PMC10164179; doi:10.1038/s41467-023-38291-1)
Supplement: Supplementary file 1 — Supplementary Information [file 41467_2023_38291_MOESM1_ESM.pdf]

Supplementary Information

Roles of adenine methylation in the physiology of *Lacticaseibacillus paracasei*
Zhao and Zhang et al.

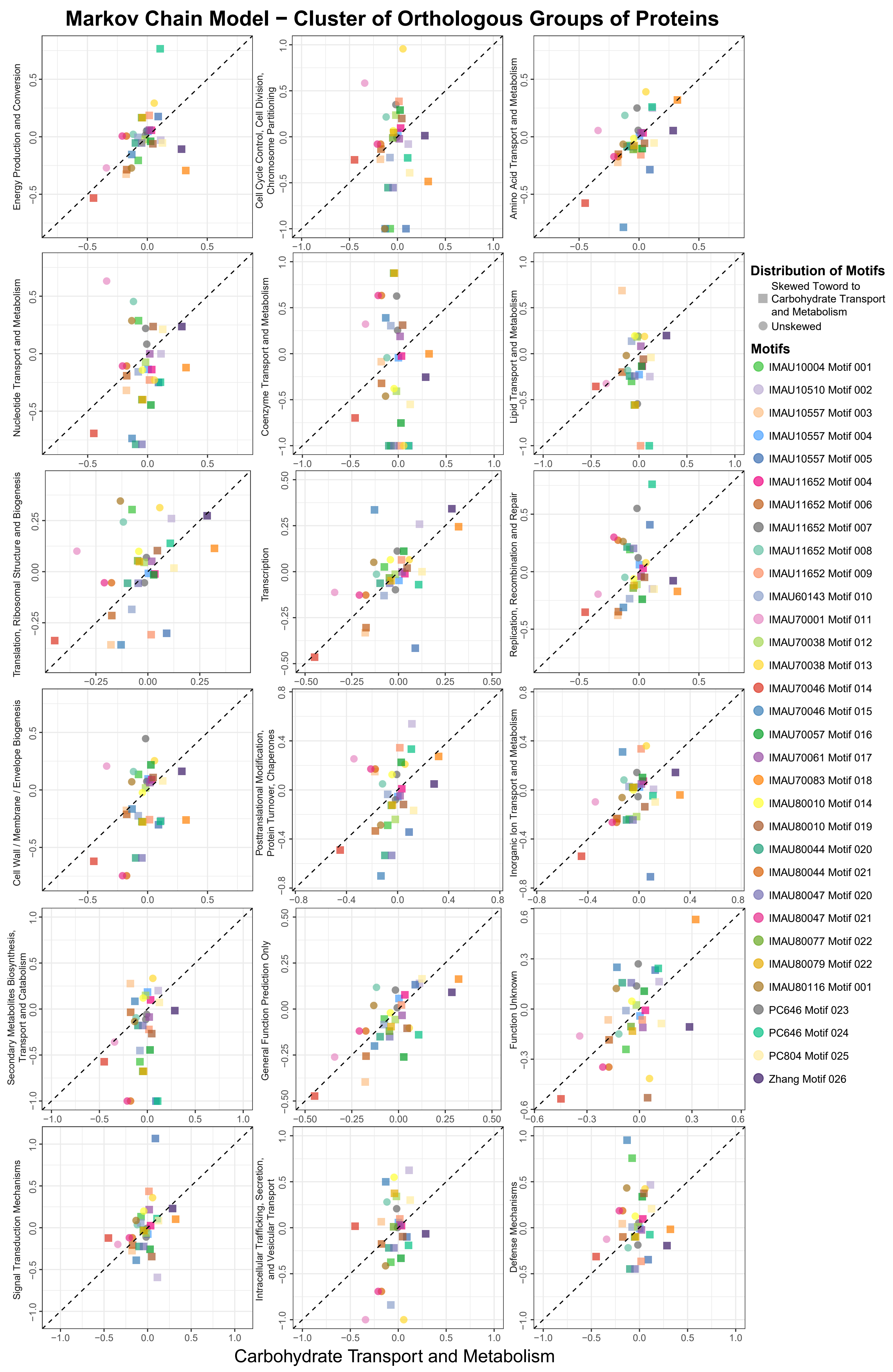
Markov Chain Model − Cluster of Orthologous Groups of Proteins
Energy Production and Conversion
Cell Cycle Control, Cell Division, Chromosome Partitioning
Amino Acid Transport and Metabolism
Nucleotide Transport and Metabolism
Coenzyme Transport and Metabolism
Lipid Transport and Metabolism
Translation, Ribosomal Structure and Biogenesis
Transcription
Replication, Recombination and Repair
Cell Wall / Membrane / Envelope Biogenesis
Posttranslational Modification, Protein Turnover, Chaperones
Inorganic Ion Transport and Metabolism
Secondary Metabolites Biosynthesis, Transport and Catabolism
General Function Prediction Only
Function Unknown
Signal Transduction Mechanisms
Intracellular Trafficking, Secretion, and Vesicular Transport
Defense Mechanisms
Carbohydrate Transport and Metabolism
Distribution of Motifs
Skewed Toword to Carbohydrate Transport and Metabolism
Unskewed
Motifs
IMAU10004 Motif 001
IMAU10510 Motif 002
IMAU10557 Motif 003
IMAU10557 Motif 004
IMAU10557 Motif 005
IMAU11652 Motif 004
IMAU11652 Motif 006
IMAU11652 Motif 007
IMAU11652 Motif 008
IMAU11652 Motif 009
IMAU60143 Motif 010
IMAU70001 Motif 011
IMAU70038 Motif 012
IMAU70038 Motif 013
IMAU70046 Motif 014
IMAU70046 Motif 015
IMAU70057 Motif 016
IMAU70061 Motif 017
IMAU70083 Motif 018
IMAU80010 Motif 014
IMAU80010 Motif 019
IMAU80044 Motif 020
IMAU80044 Motif 021
IMAU80047 Motif 020
IMAU80047 Motif 021
IMAU80077 Motif 022
IMAU80079 Motif 022
IMAU80116 Motif 001
PC646 Motif 023
PC646 Motif 024
PC804 Motif 025
Zhang Motif 026

Figure S1 Skewed distribution of methylation sites towards the coding sequences (CDS) of genes in the Clusters of Orthologous Groups of proteins (COG) functional category of carbohydrate transport and metabolism [G]. The results are generated by the Markov chain approach that considers composition. The distance between a symbol and its projection on the diagonal line measures the extent of usage biases between the COG functional category [G] and each of the other 18 COG functional categories shown. Symbols below and above the diagonal line represent skewing towards the COG functional category [G] and the COG functional category specified in each plot, respectively. Strains with more than nine (among the 18) COG functional categories located below the diagonal line are considered to have an overall skewed distribution (towards the COG functional category [G]). Each symbol represents the data of a specific motif, and each motif is represented by a different color, assigned to a specific number. Motifs of skewed and unskewed distribution are represented by a square or a circle, respectively.

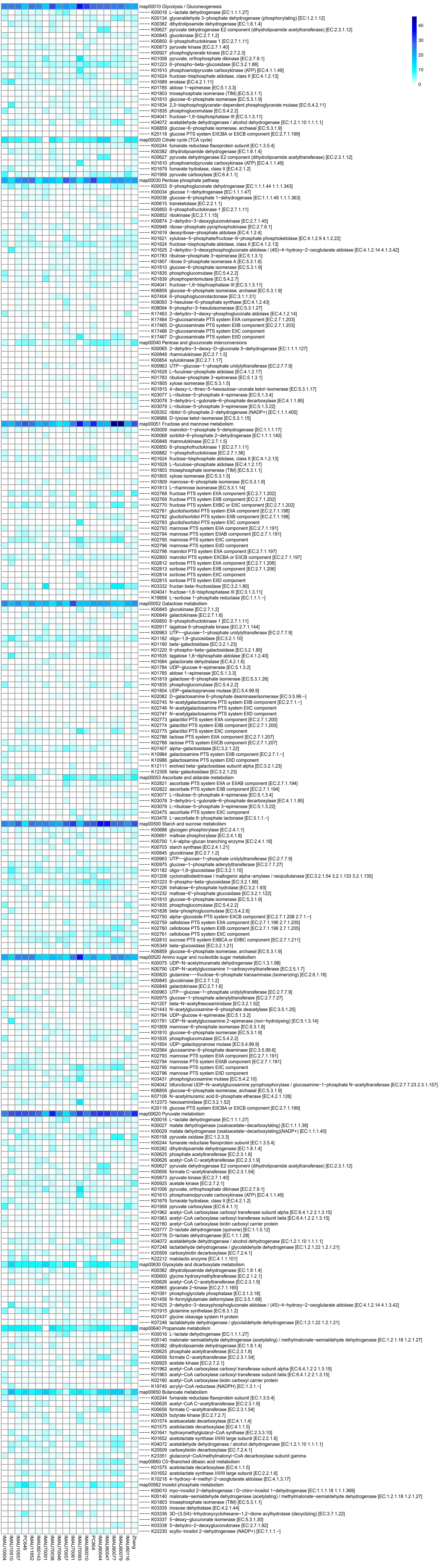
40
30
20
10
0
map00010 Glycolysis / Gluconeogenesis
---- K00016 L-lactate dehydrogenase [EC:1.1.1.27]
---- K00134 glyceraldehyde 3-phosphate dehydrogenase (phosphorylating) [EC:1.2.1.12]
---- K00382 dihydrolipoamide dehydrogenase [EC:1.8.1.4]
---- K00627 pyruvate dehydrogenase E2 component (dihydrolipoamide acetyltransferase) [EC:2.3.1.12]
---- K00845 glucokinase [EC:2.7.1.2]
---- K00850 6-phosphofructokinase 1 [EC:2.7.1.11]
---- K00873 pyruvate kinase [EC:2.7.1.40]
---- K00927 phosphoglycerate kinase [EC:2.7.2.3]
---- K01006 pyruvate, orthophosphate dikinase [EC:2.7.9.1]
---- K01223 6-phospho-beta-glucosidase [EC:3.2.1.86]
---- K01610 phosphoenolpyruvate carboxykinase (ATP) [EC:4.1.1.49]
---- K01624 fructose-bisphosphate aldolase, class II [EC:4.1.2.13]
---- K01689 enolase [EC:4.2.1.11]
---- K01785 aldose 1-epimerase [EC:5.1.3.3]
---- K01803 triosephosphate isomerase (TIM) [EC:5.3.1.1]
---- K01810 glucose-6-phosphate isomerase [EC:5.3.1.9]
---- K01834 2,3-bisphosphoglycerate-dependent phosphoglycerate mutase [EC:5.4.2.11]
---- K01835 phosphoglucomutase [EC:5.4.2.2]
---- K04041 fructose-1,6-bisphosphatase III [EC:3.1.3.11]
---- K04072 acetaldehyde dehydrogenase / alcohol dehydrogenase [EC:1.2.1.10 1.1.1.1]
---- K06859 glucose-6-phosphate isomerase, archaeal [EC:5.3.1.9]
---- K20118 glucose PTS system EIICBA or EIICB component [EC:2.7.1.199]
map00020 Citrate cycle (TCA cycle)
---- K00244 fumarate reductase flavoprotein subunit [EC:1.3.5.4]
---- K00382 dihydrolipoamide dehydrogenase [EC:1.8.1.4]
---- K00627 pyruvate dehydrogenase E2 component (dihydrolipoamide acetyltransferase) [EC:2.3.1.12]
---- K01610 phosphoenolpyruvate carboxykinase (ATP) [EC:4.1.1.49]
---- K01679 fumarate hydratase, class II [EC:4.2.1.2]
---- K01958 pyruvate carboxylase [EC:6.4.1.1]
map00030 Pentose phosphate pathway
---- K00033 6-phosphogluconate dehydrogenase [EC:1.1.1.44 1.1.1.343]
---- K00034 glucose 1-dehydrogenase [EC:1.1.1.47]
---- K00036 glucose-6-phosphate 1-dehydrogenase [EC:1.1.1.49 1.1.1.363]
---- K00615 transketolase [EC:2.2.1.1]
---- K00850 6-phosphofructokinase 1 [EC:2.7.1.11]
---- K00852 ribokinase [EC:2.7.1.15]
---- K00874 2-dehydro-3-deoxygluconokinase [EC:2.7.1.45]
---- K00948 ribose-phosphate pyrophosphokinase [EC:2.7.6.1]
---- K01619 deoxyribose-phosphate aldolase [EC:4.1.2.4]
---- K01621 xylulose-5-phosphate/fructose-6-phosphate phosphoketolase [EC:4.1.2.9 4.1.2.22]
---- K01624 fructose-bisphosphate aldolase, class II [EC:4.1.2.13]
---- K01625 2-dehydro-3-deoxyphosphogluconate aldolase / (4S)-4-hydroxy-2-oxoglutarate aldolase [EC:4.1.2.14 4.1.3.42]
---- K01783 ribulose-phosphate 3-epimerase [EC:5.1.3.1]
---- K01807 ribose 5-phosphate isomerase A [EC:5.3.1.6]
---- K01810 glucose-6-phosphate isomerase [EC:5.3.1.9]
---- K01835 phosphoglucomutase [EC:5.4.2.2]
---- K01839 phosphopentomutase [EC:5.4.2.7]
---- K04041 fructose-1,6-bisphosphatase III [EC:3.1.3.11]
---- K06859 glucose-6-phosphate isomerase, archaeal [EC:5.3.1.9]
---- K07404 6-phosphogluconolactonase [EC:3.1.1.31]
---- K08093 3-hexulose-6-phosphate synthase [EC:4.1.2.43]
---- K08094 6-phospho-3-hexuloisomerase [EC:5.3.1.27]
---- K17463 2-dehydro-3-deoxy-phosphogluconate aldolase [EC:4.1.2.14]
---- K17464 D-glucosaminate PTS system EIIA component [EC:2.7.1.203]
---- K17465 D-glucosaminate PTS system EIIB component [EC:2.7.1.203]
---- K17466 D-glucosaminate PTS system EIIC component
---- K17467 D-glucosaminate PTS system EIID component
map00040 Pentose and glucuronate interconversions
---- K00065 2-dehydro-3-deoxy-D-gluconate 5-dehydrogenase [EC:1.1.1.127]
---- K00848 rhamnulokinase [EC:2.7.1.5]
---- K00854 xylulokinase [EC:2.7.1.17]
---- K00963 UTP--glucose-1-phosphate uridylyltransferase [EC:2.7.7.9]
---- K01628 L-fuculose-phosphate aldolase [EC:4.1.2.17]
---- K01783 ribulose-phosphate 3-epimerase [EC:5.1.3.1]
---- K01805 xylose isomerase [EC:5.3.1.5]
---- K01815 4-deoxy-L-threo-5-hexosulose-uronate ketol-isomerase [EC:5.3.1.17]
---- K03077 L-ribulose-5-phosphate 4-epimerase [EC:5.1.3.4]
---- K03078 3-dehydro-L-gulonate-6-phosphate decarboxylase [EC:4.1.1.85]
---- K03079 L-ribulose-5-phosphate 3-epimerase [EC:5.1.3.22]
---- K05352 ribitol-5-phosphate 2-dehydrogenase (NADP+) [EC:1.1.1.405]
---- K09988 D-lyxose ketol-isomerase [EC:5.3.1.15]
map00051 Fructose and mannose metabolism
---- K00009 mannitol-1-phosphate 5-dehydrogenase [EC:1.1.1.17]
---- K00068 sorbitol-6-phosphate 2-dehydrogenase [EC:1.1.1.140]
---- K00848 rhamnulokinase [EC:2.7.1.5]
---- K00850 6-phosphofructokinase 1 [EC:2.7.1.11]
---- K00882 1-phosphofructokinase [EC:2.7.1.56]
---- K01624 fructose-bisphosphate aldolase, class II [EC:4.1.2.13]
---- K01628 L-fuculose-phosphate aldolase [EC:4.1.2.17]
---- K01803 triosephosphate isomerase (TIM) [EC:5.3.1.1]
---- K01805 xylose isomerase [EC:5.3.1.5]
---- K01809 mannose-6-phosphate isomerase [EC:5.3.1.8]
---- K01813 L-rhamnose isomerase [EC:5.3.1.14]
---- K02768 fructose PTS system EIIA component [EC:2.7.1.202]
---- K02769 fructose PTS system EIIB component [EC:2.7.1.202]
---- K02770 fructose PTS system EIIBC or EIIC component [EC:2.7.1.202]
---- K02781 glucitol/sorbitol PTS system EIIA component [EC:2.7.1.198]
---- K02782 glucitol/sorbitol PTS system EIIB component [EC:2.7.1.198]
---- K02783 glucitol/sorbitol PTS system EIIC component
---- K02793 mannose PTS system EIIA component [EC:2.7.1.191]
---- K02794 mannose PTS system EIIAB component [EC:2.7.1.191]
---- K02795 mannose PTS system EIIC component
---- K02796 mannose PTS system EIID component
---- K02798 mannitol PTS system EIIA component [EC:2.7.1.197]
---- K02800 mannitol PTS system EIICBA or EIICB component [EC:2.7.1.197]
---- K02812 sorbose PTS system EIIA component [EC:2.7.1.206]
---- K02813 sorbose PTS system EIIB component [EC:2.7.1.206]
---- K02814 sorbose PTS system EIIC component
---- K02815 sorbose PTS system EIID component
---- K03332 fructan beta-fructosidase [EC:3.2.1.80]
---- K04041 fructose-1,6-bisphosphatase III [EC:3.1.3.11]
---- K19956 L-sorbose 1-phosphate reductase [EC:1.1.1.-]
map00052 Galactose metabolism
---- K00845 glucokinase [EC:2.7.1.2]
---- K00849 galactokinase [EC:2.7.1.6]
---- K00850 6-phosphofructokinase 1 [EC:2.7.1.11]
---- K00917 tagatose 6-phosphate kinase [EC:2.7.1.144]
---- K00963 UTP--glucose-1-phosphate uridylyltransferase [EC:2.7.7.9]
---- K01182 oligo-1,6-glucosidase [EC:3.2.1.10]
---- K01190 beta-galactosidase [EC:3.2.1.23]
---- K01220 6-phospho-beta-galactosidase [EC:3.2.1.85]
---- K01635 tagatose 1,6-diphosphate aldolase [EC:4.1.2.40]
---- K01684 galactonate dehydratase [EC:4.2.1.6]
---- K01784 UDP-glucose 4-epimerase [EC:5.1.3.2]
---- K01785 aldose 1-epimerase [EC:5.1.3.3]
---- K01819 galactose-6-phosphate isomerase [EC:5.3.1.26]
---- K01835 phosphoglucomutase [EC:5.4.2.2]
---- K01854 UDP-galactopyranose mutase [EC:5.4.99.9]
---- K02082 D-galactosamine 6-phosphate deaminase/isomerase [EC:3.5.99.-]
---- K02745 N-acetylgalactosamine PTS system EIIB component [EC:2.7.1.-]
---- K02746 N-acetylgalactosamine PTS system EIIC component
---- K02747 N-acetylgalactosamine PTS system EIID component
---- K02773 galactitol PTS system EIIA component [EC:2.7.1.200]
---- K02774 galactitol PTS system EIIB component [EC:2.7.1.200]
---- K02775 galactitol PTS system EIIC component
---- K02786 lactose PTS system EIIA component [EC:2.7.1.207]
---- K02788 lactose PTS system EIICB component [EC:2.7.1.207]
---- K07407 alpha-galactosidase [EC:3.2.1.22]
---- K10984 galactosamine PTS system EIIB component [EC:2.7.1.-]
---- K10986 galactosamine PTS system EIID component
---- K12111 evolved beta-galactosidase subunit alpha [EC:3.2.1.23]
---- K12308 beta-galactosidase [EC:3.2.1.23]
map00053 Ascorbate and aldarate metabolism
---- K02821 ascorbate PTS system EIIA or EIIAB component [EC:2.7.1.194]
---- K02822 ascorbate PTS system EIIB component [EC:2.7.1.194]
---- K03077 L-ribulose-5-phosphate 4-epimerase [EC:5.1.3.4]
---- K03078 3-dehydro-L-gulonate-6-phosphate decarboxylase [EC:4.1.1.85]
---- K03079 L-ribulose-5-phosphate 3-epimerase [EC:5.1.3.22]
---- K03475 ascorbate PTS system EIIC component
---- K03476 L-ascorbate 6-phosphate lactonase [EC:3.1.1.-]
map00500 Starch and sucrose metabolism
---- K00688 glycogen phosphorylase [EC:2.4.1.1]
---- K00691 maltose phosphorylase [EC:2.4.1.8]
---- K00700 1,4-alpha-glucan branching enzyme [EC:2.4.1.18]
---- K00703 starch synthase [EC:2.4.1.21]
---- K00845 glucokinase [EC:2.7.1.2]
---- K00963 UTP--glucose-1-phosphate uridylyltransferase [EC:2.7.7.9]
---- K00975 glucose-1-phosphate adenylyltransferase [EC:2.7.7.27]
---- K01182 oligo-1,6-glucosidase [EC:3.2.1.10]
---- K01208 cyclomaltodextrinase / maltogenic alpha-amylase / neopullulanase [EC:3.2.1.54 3.2.1.133 3.2.1.135]
---- K01223 6-phospho-beta-glucosidase [EC:3.2.1.86]
---- K01226 trehalose-6-phosphate hydrolase [EC:3.2.1.93]
---- K01232 maltose-6'-phosphate glucosidase [EC:3.2.1.122]
---- K01810 glucose-6-phosphate isomerase [EC:5.3.1.9]
---- K01835 phosphoglucomutase [EC:5.4.2.2]
---- K01838 beta-phosphoglucomutase [EC:5.4.2.6]
---- K02750 alpha-glucoside PTS system EIICB component [EC:2.7.1.208 2.7.1.-]
---- K02759 cellobiose PTS system EIIA component [EC:2.7.1.196 2.7.1.205]
---- K02760 cellobiose PTS system EIIB component [EC:2.7.1.196 2.7.1.205]
---- K02761 cellobiose PTS system EIIC component
---- K02810 sucrose PTS system EIIBCA or EIIBC component [EC:2.7.1.211]
---- K05349 beta-glucosidase [EC:3.2.1.21]
---- K06859 glucose-6-phosphate isomerase, archaeal [EC:5.3.1.9]
map00520 Amino sugar and nucleotide sugar metabolism
---- K00075 UDP-N-acetylmuramate dehydrogenase [EC:1.3.1.98]
---- K00790 UDP-N-acetylglucosamine 1-carboxyvinyltransferase [EC:2.5.1.7]
---- K00820 glutamine---fructose-6-phosphate transaminase (isomerizing) [EC:2.6.1.16]
---- K00845 glucokinase [EC:2.7.1.2]
---- K00849 galactokinase [EC:2.7.1.6]
---- K00963 UTP--glucose-1-phosphate uridylyltransferase [EC:2.7.7.9]
---- K00975 glucose-1-phosphate adenylyltransferase [EC:2.7.7.27]
---- K01207 beta-N-acetylhexosaminidase [EC:3.2.1.52]
---- K01443 N-acetylglucosamine-6-phosphate deacetylase [EC:3.5.1.25]
---- K01784 UDP-glucose 4-epimerase [EC:5.1.3.2]
---- K01791 UDP-N-acetylglucosamine 2-epimerase (non-hydrolysing) [EC:5.1.3.14]
---- K01809 mannose-6-phosphate isomerase [EC:5.3.1.8]
---- K01810 glucose-6-phosphate isomerase [EC:5.3.1.9]
---- K01835 phosphoglucomutase [EC:5.4.2.2]
---- K01854 UDP-galactopyranose mutase [EC:5.4.99.9]
---- K02564 glucosamine-6-phosphate deaminase [EC:3.5.99.6]
---- K02793 mannose PTS system EIIA component [EC:2.7.1.191]
---- K02794 mannose PTS system EIIAB component [EC:2.7.1.191]
---- K02795 mannose PTS system EIIC component
---- K02796 mannose PTS system EIID component
---- K03431 phosphoglucosamine mutase [EC:5.4.2.10]
---- K04042 bifunctional UDP-N-acetylglucosamine pyrophosphorylase / glucosamine-1-phosphate N-acetyltransferase [EC:2.7.7.23 2.3.1.157]
---- K06859 glucose-6-phosphate isomerase, archaeal [EC:5.3.1.9]
---- K07106 N-acetylmuramic acid 6-phosphate etherase [EC:4.2.1.126]
---- K12373 hexosaminidase [EC:3.2.1.52]
---- K20118 glucose PTS system EIICBA or EIICB component [EC:2.7.1.199]
map00620 Pyruvate metabolism
---- K00016 L-lactate dehydrogenase [EC:1.1.1.27]
---- K00027 malate dehydrogenase (oxaloacetate-decarboxylating) [EC:1.1.1.38]
---- K00029 malate dehydrogenase (oxaloacetate-decarboxylating)(NADP+) [EC:1.1.1.40]
---- K00158 pyruvate oxidase [EC:1.2.3.3]
---- K00244 fumarate reductase flavoprotein subunit [EC:1.3.5.4]
---- K00382 dihydrolipoamide dehydrogenase [EC:1.8.1.4]
---- K00625 phosphate acetyltransferase [EC:2.3.1.8]
---- K00626 acetyl-CoA C-acetyltransferase [EC:2.3.1.9]
---- K00627 pyruvate dehydrogenase E2 component (dihydrolipoamide acetyltransferase) [EC:2.3.1.12]
---- K00656 formate C-acetyltransferase [EC:2.3.1.54]
---- K00873 pyruvate kinase [EC:2.7.1.40]
---- K00925 acetate kinase [EC:2.7.2.1]
---- K01006 pyruvate, orthophosphate dikinase [EC:2.7.9.1]
---- K01610 phosphoenolpyruvate carboxykinase (ATP) [EC:4.1.1.49]
---- K01679 fumarate hydratase, class II [EC:4.2.1.2]
---- K01958 pyruvate carboxylase [EC:6.4.1.1]
---- K01962 acetyl-CoA carboxylase carboxyl transferase subunit alpha [EC:6.4.1.2 2.1.3.15]
---- K01963 acetyl-CoA carboxylase carboxyl transferase subunit beta [EC:6.4.1.2 2.1.3.15]
---- K02160 acetyl-CoA carboxylase biotin carboxyl carrier protein
---- K03777 D-lactate dehydrogenase (quinone) [EC:1.1.5.12]
---- K03778 D-lactate dehydrogenase [EC:1.1.1.28]
---- K04072 acetaldehyde dehydrogenase / alcohol dehydrogenase [EC:1.2.1.10 1.1.1.1]
---- K07248 lactaldehyde dehydrogenase / glycolaldehyde dehydrogenase [EC:1.2.1.22 1.2.1.21]
---- K20509 carboxybiotin decarboxylase [EC:7.2.4.1]
---- K22212 malolactic enzyme [EC:4.1.1.101]
map00630 Glyoxylate and dicarboxylate metabolism
---- K00382 dihydrolipoamide dehydrogenase [EC:1.8.1.4]
---- K00600 glycine hydroxymethyltransferase [EC:2.1.2.1]
---- K00626 acetyl-CoA C-acetyltransferase [EC:2.3.1.9]
---- K00865 glycerate 2-kinase [EC:2.7.1.165]
---- K01091 phosphoglycolate phosphatase [EC:3.1.3.18]
---- K01458 N-formylglutamate deformylase [EC:3.5.1.68]
---- K01625 2-dehydro-3-deoxyphosphogluconate aldolase / (4S)-4-hydroxy-2-oxoglutarate aldolase [EC:4.1.2.14 4.1.3.42]
---- K01915 glutamine synthetase [EC:6.3.1.2]
---- K02437 glycine cleavage system H protein
---- K07248 lactaldehyde dehydrogenase / glycolaldehyde dehydrogenase [EC:1.2.1.22 1.2.1.21]
map00640 Propanoate metabolism
---- K00016 L-lactate dehydrogenase [EC:1.1.1.27]
---- K00140 malonate-semialdehyde dehydrogenase (acetylating) / methylmalonate-semialdehyde dehydrogenase [EC:1.2.1.18 1.2.1.27]
---- K00382 dihydrolipoamide dehydrogenase [EC:1.8.1.4]
---- K00625 phosphate acetyltransferase [EC:2.3.1.8]
---- K00656 formate C-acetyltransferase [EC:2.3.1.54]
---- K00925 acetate kinase [EC:2.7.2.1]
---- K01962 acetyl-CoA carboxylase carboxyl transferase subunit alpha [EC:6.4.1.2 2.1.3.15]
---- K01963 acetyl-CoA carboxylase carboxyl transferase subunit beta [EC:6.4.1.2 2.1.3.15]
---- K02160 acetyl-CoA carboxylase biotin carboxyl carrier protein
---- K19745 acryloyl-CoA reductase (NADPH) [EC:1.3.1.-]
map00650 Butanoate metabolism
---- K00244 fumarate reductase flavoprotein subunit [EC:1.3.5.4]
---- K00626 acetyl-CoA C-acetyltransferase [EC:2.3.1.9]
---- K00656 formate C-acetyltransferase [EC:2.3.1.54]
---- K00929 butyrate kinase [EC:2.7.2.7]
---- K01574 acetoacetate decarboxylase [EC:4.1.1.4]
---- K01575 acetolactate decarboxylase [EC:4.1.1.5]
---- K01641 hydroxymethylglutaryl-CoA synthase [EC:2.3.3.10]
---- K01652 acetolactate synthase I/II/III large subunit [EC:2.2.1.6]
---- K04072 acetaldehyde dehydrogenase / alcohol dehydrogenase [EC:1.2.1.10 1.1.1.1]
---- K20509 carboxybiotin decarboxylase [EC:7.2.4.1]
---- K23351 glutaconyl-CoA/methylmalonyl-CoA decarboxylase subunit gamma
map00660 C5-Branched dibasic acid metabolism
---- K01575 acetolactate decarboxylase [EC:4.1.1.5]
---- K01652 acetolactate synthase I/II/III large subunit [EC:2.2.1.6]
---- K10218 4-hydroxy-4-methyl-2-oxoglutarate aldolase [EC:4.1.3.17]
map00562 Inositol phosphate metabolism
---- K00010 myo-inositol 2-dehydrogenase / D-chiro-inositol 1-dehydrogenase [EC:1.1.1.18 1.1.1.369]
---- K00140 malonate-semialdehyde dehydrogenase (acetylating) / methylmalonate-semialdehyde dehydrogenase [EC:1.2.1.18 1.2.1.27]
---- K01803 triosephosphate isomerase (TIM) [EC:5.3.1.1]
---- K03335 inosose dehydratase [EC:4.2.1.44]
---- K03336 3D-(3,5/4)-trihydroxycyclohexane-1,2-dione acylhydrolase (decyclizing) [EC:3.7.1.22]
---- K03337 5-deoxy-glucuronate isomerase [EC:5.3.1.30]
---- K03338 5-dehydro-2-deoxygluconokinase [EC:2.7.1.92]
---- K22230 scyllo-inositol 2-dehydrogenase (NADP+) [EC:1.1.1.-]
IMAU10004
IMAU10510
IMAU10557
PC646
IMAU11652
IMAU60143
IMAU70001
IMAU70038
IMAU70046
IMAU70057
IMAU70061
IMAU70083
IMAU80010
PC804
IMAU80044
IMAU80047
IMAU80077
IMAU80079
IMAU80116
Zhang

Figure S2 Heatmap showing the distribution of methylation sites across coding sequences of 20 *Lacticaseibacillus paracasei* isolates, annotated based on the Kyoto Encyclopedia of Genes and Genomes (KEGG) database. X-axis shows the isolate number; y-axis indicates the specific KEGG category. The color scale represents the percentage of methylation sites in a specific KEGG Orthology (KO), calculated as: the number of methylation sites within the coding sequences of a specific KO divided by the total number of methylation sites across all KOs. The quotient was converted to permillage. The color bar represents the relative distribution of m6A methylation in various KOs.

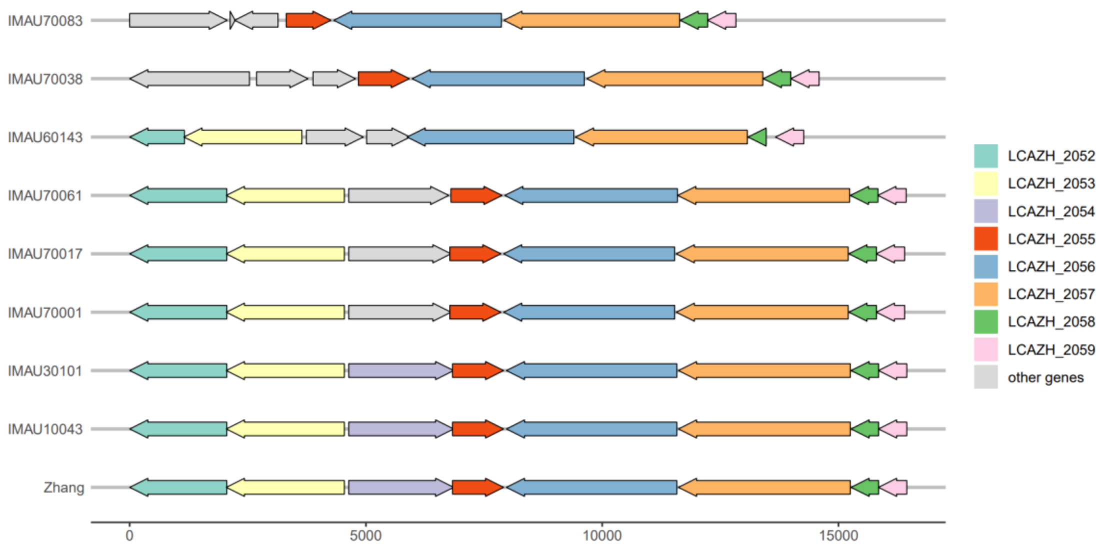

Figure S3 Comparative analysis of the gene organization of the bacteriophage exclusion (BREX) system in various *Lacticaseibacillus paracasei* strains. A complete or partial BREX gene cluster was detected in nine of the 28 investigated strains. A complete BREX system cassette in this species (previously identified in *Lacticaseibacillus paracasei* Zhang) includes eight genes, namely LCAZH_2052 (protease Lon-related BREX system protein *BrxL*, *brxL*), LCAZH_2053 (phosphatase PglZ type A, *pglZ*), LCAZH_2054 (a putative DNA MTase), LCAZH_2055 (site-specific integrase, *int*), LCAZH_2056 (adenine-specific DNA-methyltransferase, *pglX*), LCAZH_2057 (P-loop protein *BrxC*, *brxC*), LCAZH_2058 (DUF1788 domain-containing protein, *brxB*), and LCAZH_2059 (DUF1819 family protein, *brxA*). Colored arrows represent different genes, and the arrow head represents gene orientation. The scale at the bottom indicates gene length (bp).

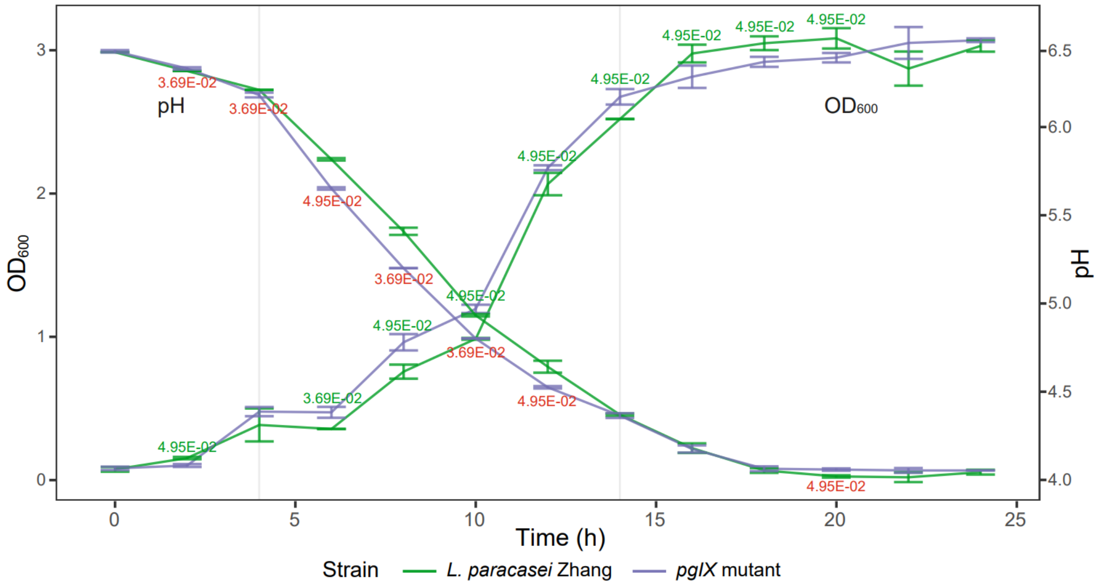

Figure S4 Growth curves of *Lacticaseibacillus* (*L.*) *paracasei* Zhang wild type (n = 3) and *pglX* mutant (n = 3), determined by changes in optical density at 600 nm (OD_{600}) and pH of the bacterial cultures over time. X-axis indicates different time points; y-axis indicates OD_{600} and pH. The logarithmic growth phase (from 4 h to 14 h) is framed by two vertical lines. Error bars represent SD; and the measure of centre for the error bars represents the mean. Statistical difference was tested by the Wilcoxon rank-sum test (two-sided). The *P* values less than 0.05 are labeled. All exact *P* values are provided in the data source file.

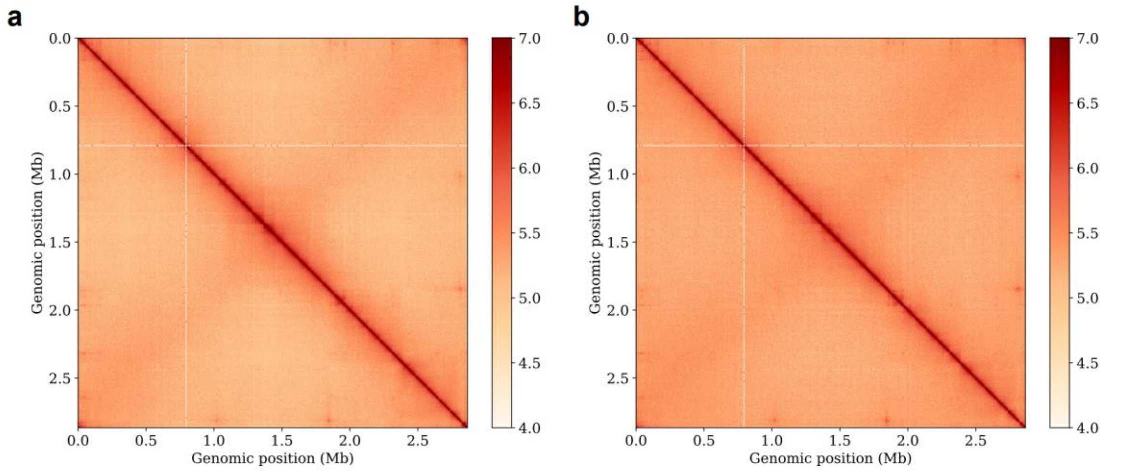

Figure S5 High-throughput chromosome conformation capture (Hi-C) contact maps of *Lacticaseibacillus paracasei* Zhang *pglX* mutant (a) and wild type (b). Both x- and y-axes show the position in the reference genome (Mb). The color bar indicates the interaction intensity; and the greater the numerical value, the stronger the interaction.

Table S1 General information of methyltransferases in 28 *Lacticaseibacillus paracasei* isolates.

Isolate	Query ID	Global identity	E-value	Bit score	REBASE:EnzType
IMAU10004	IMAU10004_01400	97.70	0	611	EnzType:putative Type II methyltransferase
IMAU10004	IMAU10004_01689	100.00	0	3207	EnzType:putative Type IIG restriction enzyme/methyltransferase
IMAU10004	IMAU10004_02169	98.17	0	1429	EnzType:putative Type IV methyl-directed restriction enzyme
IMAU10004	IMAU10004_02170	97.66	0	775	EnzType:putative Type I specificity subunit
IMAU10004	IMAU10004_02172	74.74	5.41E-180	509	EnzType:putative Type I specificity subunit
IMAU10004	IMAU10004_02173	100.00	0	1100	EnzType:putative Type I methyltransferase
IMAU10004	IMAU10004_02174	100.00	0	2109	EnzType:putative Type I restriction enzyme
IMAU10043	IMAU10043_01924	99.86	0	1519	EnzType:putative Type IIG restriction enzyme/methyltransferase
IMAU10043	IMAU10043_01926	100.00	0	2487	EnzType:Type II methyltransferase
IMAU10510	IMAU10510_02100	92.69	0	1346	EnzType:putative Type IV methyl-directed restriction enzyme
IMAU10510	IMAU10510_02102	96.94	2.44E-136	382	EnzType:putative Type I specificity subunit
IMAU10510	IMAU10510_02104	100.00	0	813	EnzType:putative Type I specificity subunit
IMAU10510	IMAU10510_02105	100.00	0	1101	EnzType:putative Type I methyltransferase
IMAU10510	IMAU10510_02106	100.00	0	2112	EnzType:putative Type I restriction enzyme
IMAU10557	IMAU10557_00932	100.00	0	801	EnzType:putative Type II restriction enzyme
IMAU10557	IMAU10557_00934	86.73	0	790	EnzType:putative Type II methyltransferase
IMAU10557	IMAU10557_01985	93.27	0	634	EnzType:putative Type II methyltransferase
IMAU10557	IMAU10557_02103	51.63	1.19E-128	367	EnzType:putative Type II methyltransferase
IMAU10557	IMAU10557_02239	100.00	0	1452	EnzType:putative Type IV methyl-directed restriction enzyme
IMAU10557	IMAU10557_02240	93.78	0	762	EnzType:putative Type I specificity subunit
IMAU10557	IMAU10557_02242	73.49	1.78E-113	323	EnzType:putative Type I specificity subunit
IMAU10557	IMAU10557_02243	100.00	0	1101	EnzType:putative Type I methyltransferase
IMAU10557	IMAU10557_02244	100.00	0	2112	EnzType:putative Type I restriction enzyme

IMAU10557	IMAU10557_03106	99.03	0	640	EnzType:putative Type II restriction enzyme
IMAU10557	IMAU10557_03107	98.42	0	1026	EnzType:putative Type II methyltransferase
IMAU10557	IMAU10557_03183	99.03	0	640	EnzType:putative Type II restriction enzyme
IMAU10557	IMAU10557_03184	95.86	0	1001	EnzType:putative Type II methyltransferase
IMAU10685	IMAU10685_02033	77.95	0	610	EnzType:putative Type I specificity subunit
IMAU10685	IMAU10685_02035	54.43	1.51E-157	445	EnzType:putative Type I specificity subunit
IMAU10685	IMAU10685_02037	83.25	4.42E-125	353	EnzType:putative Type I specificity subunit
IMAU10685	IMAU10685_02038	100.00	0	1100	EnzType:putative Type I methyltransferase
IMAU10685	IMAU10685_02039	99.70	0	2104	EnzType:putative Type I restriction enzyme
IMAU10685	IMAU10685_02919	51.09	0	1106	EnzType:putative Type I restriction enzyme
IMAU10685	IMAU10685_02920	97.96	0	1095	EnzType:putative Type I methyltransferase
IMAU10685	IMAU10685_02921	72.44	0	536	EnzType:putative Type I specificity subunit
PC646	IMAU11646_02220	99.58	0	1446	EnzType:putative Type IV methyl-directed restriction enzyme
PC646	IMAU11646_02221	67.82	4.53E-170	483	EnzType:putative Type I specificity subunit
PC646	IMAU11646_02223	75.06	3.09E-175	496	EnzType:putative Type I specificity subunit
PC646	IMAU11646_02224	100.00	0	1099	EnzType:putative Type I methyltransferase
PC646	IMAU11646_02225	100.00	0	2112	EnzType:putative Type I restriction enzyme
PC646	IMAU11646_03112	52.89	7.06E-147	417	EnzType:putative Type II methyltransferase
PC646	IMAU11646_03125	72.96	2.71E-180	509	EnzType:Type I specificity subunit
PC646	IMAU11646_03126	99.07	0	1108	EnzType:putative Type I methyltransferase
PC646	IMAU11646_03127	98.96	0	2160	EnzType:putative Type I restriction enzyme
IMAU11652	IMAU11652_00589	98.70	0	1104	EnzType:putative Type I methyltransferase
IMAU11652	IMAU11652_00590	99.34	0	2163	EnzType:putative Type I restriction enzyme
IMAU11652	IMAU11652_00914	99.74	0	800	EnzType:putative Type II restriction enzyme
IMAU11652	IMAU11652_00915	102.06	0	899	EnzType:putative Type II methyltransferase
IMAU11652	IMAU11652_01218	52.32	1.85E-53	166	EnzType:putative Type II methyltransferase

IMAU11652	IMAU11652_02049	100.00	4.99E-176	486	EnzType:putative Type II methyltransferase
IMAU11652	IMAU11652_02050	100.00	0	3046	EnzType:putative Type IIG restriction enzyme/methyltransferase
IMAU11652	IMAU11652_02168	99.58	0	1447	EnzType:putative Type IV methyl-directed restriction enzyme
IMAU11652	IMAU11652_02169	75.69	0	550	EnzType:putative Type I specificity subunit
IMAU11652	IMAU11652_02171	79.74	3.63E-174	494	EnzType:putative Type I specificity subunit
IMAU11652	IMAU11652_02172	99.25	0	1093	EnzType:putative Type I methyltransferase
IMAU11652	IMAU11652_02173	99.80	0	2105	EnzType:putative Type I restriction enzyme
IMAU11652	IMAU11652_02997	67.92	0	1209	EnzType:putative Type III restriction enzyme
IMAU11652	IMAU11652_02998	72.02	0	945	EnzType:putative Type III methyltransferase
IMAU30101	IMAU30101_01926	99.86	0	1519	EnzType:putative Type IIG restriction enzyme/methyltransferase
IMAU30101	IMAU30101_01928	100.00	0	2487	EnzType:Type II methyltransferase
IMAU60143	IMAU60143_00356	100.00	0	513	EnzType:putative Type II methyltransferase
IMAU60143	IMAU60143_01813	91.74	0	709	EnzType:putative Type II restriction enzyme
IMAU60143	IMAU60143_01814	87.42	0	601	EnzType:putative Type II methyltransferase
IMAU60143	IMAU60143_01852	98.68	1.32E-109	311	EnzType:putative Type II methyltransferase
IMAU60143	IMAU60143_02004	73.33	4.66E-172	482	EnzType:putative Type IV methyl-directed restriction enzyme
IMAU60143	IMAU60143_02010	79.28	0	1922	EnzType:putative Type II methyltransferase
IMAU60143	IMAU60143_02228	98.68	1.32E-109	311	EnzType:putative Type II methyltransferase
IMAU70001	IMAU70001_01390	97.70	0	611	EnzType:putative Type II methyltransferase
IMAU70001	IMAU70001_01684	84.02	0	2704	EnzType:putative Type IIG restriction enzyme/methyltransferase
IMAU70001	IMAU70001_01694	84.02	0	2697	EnzType:putative Type IIG restriction enzyme/methyltransferase
IMAU70001	IMAU70001_02182	89.68	0	2141	EnzType:putative Type II methyltransferase
IMAU70001	IMAU70001_02631	99.56	4.49E-170	469	EnzType:putative Type II methyltransferase
IMAU70001	IMAU70001_02675	90.23	0	1769	EnzType:putative Type IIG restriction enzyme/methyltransferase
IMAU70017	IMAU70017_01372	97.70	0	611	EnzType:putative Type II methyltransferase
IMAU70017	IMAU70017_01665	84.02	0	2701	EnzType:putative Type IIG restriction enzyme/methyltransferase

IMAU70017	IMAU70017_02154	89.68	0	2141	EnzType:putative Type II methyltransferase
IMAU70018	IMAU70018_01630	98.55	0	560	EnzType:putative Type II methyltransferase
PC724	IMAU70024_02098	98.92	0	565	EnzType:putative Type I specificity subunit
PC724	IMAU70024_02102	99.44	0	1093	EnzType:putative Type I methyltransferase
PC724	IMAU70024_02103	99.41	0	2099	EnzType:putative Type I restriction enzyme
IMAU70027	IMAU70027_01671	66.54	0	2165	EnzType:putative Type IIG restriction enzyme/methyltransferase
IMAU70027	IMAU70027_02208	98.17	0	1429	EnzType:putative Type IV methyl-directed restriction enzyme
IMAU70027	IMAU70027_02209	96.10	0	866	EnzType:putative Type I specificity subunit
IMAU70027	IMAU70027_02211	79.12	0	637	EnzType:putative Type I specificity subunit
IMAU70027	IMAU70027_02214	85.53	0	944	EnzType:putative Type I methyltransferase
IMAU70027	IMAU70027_02216	100.00	0	2109	EnzType:putative Type I restriction enzyme
IMAU70038	IMAU70038_00491	87.82	0	723	EnzType:putative Type II methyltransferase
IMAU70038	IMAU70038_00492	81.64	0	696	EnzType:putative Type II restriction enzyme
IMAU70038	IMAU70038_01968	79.92	4.08E-147	411	EnzType:putative Type II methyltransferase
IMAU70038	IMAU70038_02006	93.08	0	2353	EnzType:putative Type IIG restriction enzyme/methyltransferase
IMAU70038	IMAU70038_02146	98.31	0	1432	EnzType:putative Type IV methyl-directed restriction enzyme
IMAU70038	IMAU70038_02147	95.08	0	754	EnzType:putative Type I specificity subunit
IMAU70038	IMAU70038_02149	71.57	5.72E-172	489	EnzType:putative Type I specificity subunit
IMAU70038	IMAU70038_02150	99.81	0	1098	EnzType:putative Type I methyltransferase
IMAU70038	IMAU70038_02151	100.00	0	2109	EnzType:putative Type I restriction enzyme
IMAU70046	IMAU70046_01967	96.06	0	1400	EnzType:putative Type IV methyl-directed restriction enzyme
IMAU70046	IMAU70046_01968	56.09	2.95E-156	447	EnzType:putative Type I specificity subunit
IMAU70046	IMAU70046_01970	75.06	2.53E-160	459	EnzType:putative Type I specificity subunit
IMAU70046	IMAU70046_01971	100.00	0	1126	EnzType:putative Type I methyltransferase
IMAU70046	IMAU70046_01972	98.16	0	2105	EnzType:putative Type I restriction enzyme
IMAU70046	IMAU70046_02430	100.00	0	513	EnzType:putative Type II methyltransferase

IMAU70046	IMAU70046_02782	98.67	0	2154	EnzType:putative Type I restriction enzyme
IMAU70046	IMAU70046_02783	98.70	0	1106	EnzType:putative Type I methyltransferase
IMAU70046	IMAU70046_02784	82.35	5.01E-174	495	EnzType:putative Type I specificity subunit
IMAU70057	IMAU70057_01997	100.00	4.99E-176	486	EnzType:putative Type II methyltransferase
IMAU70057	IMAU70057_01999	91.33	0	2786	EnzType:putative Type IIG restriction enzyme/methyltransferase
IMAU70057	IMAU70057_02126	99.86	0	1450	EnzType:putative Type IV methyl-directed restriction enzyme
IMAU70057	IMAU70057_02127	60.71	1.43E-83	245	EnzType:putative Type I specificity subunit
IMAU70057	IMAU70057_02128	59.12	1.09E-175	492	EnzType:putative Type I specificity subunit
IMAU70057	IMAU70057_02130	73.13	0	515	EnzType:putative Type I specificity subunit
IMAU70057	IMAU70057_02131	100.00	0	1101	EnzType:putative Type I methyltransferase
IMAU70057	IMAU70057_02132	100.00	0	2112	EnzType:putative Type I restriction enzyme
IMAU70057	IMAU70057_03015	57.10	0	1114	EnzType:putative Type IIG restriction enzyme/methyltransferase
IMAU70061	IMAU70061_02182	85.69	0	2065	EnzType:putative Type II methyltransferase
IMAU70061	IMAU70061_02400	90.12	1.14E-108	309	EnzType:putative Type II methyltransferase
IMAU70061	IMAU70061_02401	70.00	2.65E-130	372	EnzType:putative Type II methyltransferase
IMAU70061	IMAU70061_02606	93.03	3.36E-178	492	EnzType:putative Type II methyltransferase
IMAU70061	IMAU70061_02618	99.70	0	2036	EnzType:putative Type III restriction enzyme
IMAU70061	IMAU70061_02619	99.52	0	1281	EnzType:putative Type III methyltransferase
IMAU70061	IMAU70061_02622	59.70	7.54E-24	88.2	EnzType:putative control protein
IMAU70083	IMAU70083_02830	100.00	0	699	EnzType:putative Type II methyltransferase
IMAU70083	IMAU70083_02860	93.44	5.85E-173	479	EnzType:putative Type II methyltransferase
IMAU70083	IMAU70083_02888	73.80	0	1039	EnzType:putative Type IIG restriction enzyme/methyltransferase
IMAU70083	IMAU70083_02892	86.70	0	2065	EnzType:putative Type IIG restriction enzyme/methyltransferase
IMAU70083	IMAU70083_03037	100.00	0	1452	EnzType:putative Type IV methyl-directed restriction enzyme
IMAU70083	IMAU70083_03038	100.00	0	784	EnzType:putative Type I specificity subunit
IMAU70083	IMAU70083_03040	69.55	0	543	EnzType:putative Type I specificity subunit

IMAU70083	IMAU70083_03041	100.00	0	1101	EnzType:putative Type I methyltransferase
IMAU70083	IMAU70083_03042	100.00	0	2112	EnzType:putative Type I restriction enzyme
IMAU80010	IMAU80010_00598	61.54	6.76E-164	469	EnzType:putative Type I specificity subunit
IMAU80010	IMAU80010_00599	99.63	0	1112	EnzType:putative Type I methyltransferase
IMAU80010	IMAU80010_00600	98.67	0	2154	EnzType:putative Type I restriction enzyme
IMAU80010	IMAU80010_01951	99.60	0	530	EnzType:putative Type II methyltransferase
IMAU80010	IMAU80010_02191	98.03	0	1427	EnzType:putative Type IV methyl-directed restriction enzyme
IMAU80010	IMAU80010_02192	72.85	0	568	EnzType:putative Type I specificity subunit
IMAU80010	IMAU80010_02194	95.59	0	800	EnzType:putative Type I specificity subunit
IMAU80010	IMAU80010_02195	100.00	0	1127	EnzType:putative Type I methyltransferase
IMAU80010	IMAU80010_02196	98.83	0	2116	EnzType:putative Type I restriction enzyme
PC804	IMAU80040_00345	75.59	0	1590	EnzType:putative Type I restriction enzyme
PC804	IMAU80040_00346	100.00	0	1100	EnzType:putative Type I methyltransferase
PC804	IMAU80040_00347	50.94	4.85E-96	280	EnzType:putative Type I specificity subunit
PC804	IMAU80040_00352	95.83	0	805	EnzType:putative Type I specificity subunit
PC804	IMAU80040_00353	98.31	0	1432	EnzType:putative Type IV methyl-directed restriction enzyme
PC804	IMAU80040_00475	91.33	0	2788	EnzType:putative Type IIG restriction enzyme/methyltransferase
PC804	IMAU80040_00477	100.00	4.99E-176	486	EnzType:putative Type II methyltransferase
PC804	IMAU80040_02981	99.66	0	2436	EnzType:putative Type IIG restriction enzyme/methyltransferase
IMAU80044	IMAU80044_01670	94.88	0	1923	EnzType:putative Type IIG restriction enzyme/methyltransferase
IMAU80044	IMAU80044_02167	75.25	1.58E-178	506	EnzType:putative Type I specificity subunit
IMAU80044	IMAU80044_02169	95.14	0	759	EnzType:putative Type I specificity subunit
IMAU80044	IMAU80044_02170	100.00	0	1100	EnzType:putative Type I methyltransferase
IMAU80044	IMAU80044_02171	100.00	0	2108	EnzType:putative Type I restriction enzyme
IMAU80047	IMAU80047_01669	94.88	0	1923	EnzType:putative Type IIG restriction enzyme/methyltransferase
IMAU80047	IMAU80047_02165	75.25	1.58E-178	506	EnzType:putative Type I specificity subunit

IMAU80047	IMAU80047_02167	95.14	0	759	EnzType:putative Type I specificity subunit
IMAU80047	IMAU80047_02168	100.00	0	1100	EnzType:putative Type I methyltransferase
IMAU80047	IMAU80047_02169	100.00	0	2108	EnzType:putative Type I restriction enzyme
IMAU80048	IMAU80048_01446	75.59	0	1590	EnzType:putative Type I restriction enzyme
IMAU80048	IMAU80048_01447	100.00	0	1100	EnzType:putative Type I methyltransferase
IMAU80048	IMAU80048_01451	68.06	7.82E-162	461	EnzType:putative Type I specificity subunit
IMAU80048	IMAU80048_01453	98.31	0	1432	EnzType:putative Type IV methyl-directed restriction enzyme
IMAU80048	IMAU80048_01561	91.33	0	2788	EnzType:putative Type IIG restriction enzyme/methyltransferase
IMAU80048	IMAU80048_01563	100.00	4.99E-176	486	EnzType:putative Type II methyltransferase
IMAU80077	IMAU80077_02185	59.41	5.62E-81	239	EnzType:putative Type I specificity subunit
IMAU80077	IMAU80077_02187	97.02	0	797	EnzType:putative Type I specificity subunit
IMAU80077	IMAU80077_02188	100.00	0	1100	EnzType:putative Type I methyltransferase
IMAU80077	IMAU80077_02189	99.80	0	2104	EnzType:putative Type I restriction enzyme
IMAU80077	IMAU80077_02667	99.59	0	513	EnzType:putative Type II methyltransferase
IMAU80077	IMAU80077_02701	66.12	0	1299	EnzType:putative Type IIG restriction enzyme/methyltransferase
IMAU80079	IMAU80079_02188	59.41	5.62E-81	239	EnzType:putative Type I specificity subunit
IMAU80079	IMAU80079_02190	97.02	0	797	EnzType:putative Type I specificity subunit
IMAU80079	IMAU80079_02191	100.00	0	1100	EnzType:putative Type I methyltransferase
IMAU80079	IMAU80079_02192	99.80	0	2104	EnzType:putative Type I restriction enzyme
IMAU80079	IMAU80079_02672	99.59	0	513	EnzType:putative Type II methyltransferase
IMAU80079	IMAU80079_02704	66.12	0	1299	EnzType:putative Type IIG restriction enzyme/methyltransferase
IMAU80116	IMAU80116_00959	89.45	0	511	EnzType:putative Type II methyltransferase
IMAU80116	IMAU80116_01342	97.70	0	611	EnzType:putative Type II methyltransferase
IMAU80116	IMAU80116_01634	99.94	0	3207	EnzType:putative Type IIG restriction enzyme/methyltransferase
IMAU80116	IMAU80116_02175	58.93	0	848	EnzType:putative Type IV methyl-directed restriction enzyme
IMAU80116	IMAU80116_02176	97.66	0	775	EnzType:putative Type I specificity subunit

IMAU80116	IMAU80116_02178	74.74	5.41E-180	509	EnzType:putative Type I specificity subunit
IMAU80116	IMAU80116_02179	100.00	0	1100	EnzType:putative Type I methyltransferase
IMAU80116	IMAU80116_02180	99.90	0	2107	EnzType:putative Type I restriction enzyme
IMAU80116	IMAU80116_02703	52.05	3.33E-91	266	EnzType:putative Type II methyltransferase
Zhang	LCAZH_2054	99.86	0	1519	EnzType:putative Type IIG restriction enzyme/methyltransferase
Zhang	LCAZH_2056	100.00	0	2487	EnzType:Type II methyltransferase

Table S2 Differentially expressed genes in *pglX* mutant in comparison with its wild type.

Gene locus	Gene description	Fold change	Gene name
LCAZH_0009	deoxyribose-phosphate aldolase	1.57	deoC
LCAZH_0020	metal-dependent membrane protease	1.63	-
LCAZH_0022	hypothetical protein	2.10	-
LCAZH_0057	metal-dependent membrane protease	1.80	-
LCAZH_0058	2-keto-3-deoxy-6-phosphogluconate aldolase	3.51	eda
LCAZH_0060	hypothetical protein	2.26	-
LCAZH_0061	hypothetical protein	2.25	-
LCAZH_0063	multidrug ABC transporter ATPase/permease	1.03	-
LCAZH_0095	hypothetical protein	1.18	-
LCAZH_0096	hypothetical protein	1.05	
LCAZH_0097	ABC transporter ATP-binding protein	1.18	ABC-2.A
LCAZH_0117	phospholipase A2 family enzyme	1.57	-
LCAZH_0118	hypothetical protein	1.56	-
LCAZH_0187	helicase subunit of the Holliday junction resolvase-like ATPase	1.47	ycaJ
LCAZH_0188	acetate kinase	1.32	ackA
LCAZH_0190	hypothetical protein	2.02	-
LCAZH_0191	hypothetical protein	2.14	fbaA
LCAZH_0192	PTS system ascorbate-specific transporter subunit IIC	1.86	ulaA
LCAZH_0218	flavodoxin	1.25	-
LCAZH_0257	inositol dehydrogenase	1.11	iolG
LCAZH_0264	H+/gluconate symporter-like permease	1.72	TC.GNTP
LCAZH_0311	lacto-N-biosidase	2.89	HEXA_B
LCAZH_0330	hypothetical protein	1.33	patB
LCAZH_0331	peptidase	1.63	pepP
LCAZH_0332	PTS system transporter subunit IIc	1.72	celB
LCAZH_0335	endoglucanase	2.11	-
LCAZH_0336	transcription antiterminator	2.49	licR
LCAZH_0341	hypothetical protein	1.85	-
LCAZH_0353	hypothetical protein	1.03	-
LCAZH_0357	ribose/xylose/arabinose/galactoside ABC transporter permease	1.85	rbsC
LCAZH_0358	sugar ABC transporter periplasmic protein	1.63	rbsB
LCAZH_0371	hypothetical protein	1.03	-
LCAZH_0377	transcriptional antiterminator and PTS system component IIA	2.14	-
LCAZH_0378	PTS system galactitol-specific transporter subunit IIB	2.20	ulaB
LCAZH_0379	hypothetical protein	2.08	ulaA

LCAZH_0380	hypothetical protein	2.93	-
LCAZH_0381	fructose/tagatose bisphosphate aldolase	1.94	fbaA
LCAZH_0402	levA protein	4.11	agaF
LCAZH_0403	PTS system mannose/fructose/N-acetylgalactosamine-specific transporter subunit IIB	3.92	manX
LCAZH_0404	levC protein	3.89	manY
LCAZH_0405	PTS system mannose/fructose/N-acetylgalactosamine-specific transporter subunit IID	4.02	-
LCAZH_0406	hypothetical protein	4.05	-
LCAZH_0407	fructan hydrolase	5.16	fruA
LCAZH_0413	6-phospho 3-hexuloisomerase	1.27	hxlB
LCAZH_0449	oxidoreductase	1.14	ohyA
LCAZH_0456	redox protein%2C regulator of disulfide bond formation	1.41	osmC
LCAZH_0540	hypothetical protein	1.28	-
LCAZH_0542	hypothetical protein	1.08	-
LCAZH_0554	l-lactate dehydrogenase	2.10	ldh
LCAZH_0559	redox protein%2C regulator of disulfide bond formation	1.68	osmC
LCAZH_0603	PTS system galacitol-specific transporter subunit IIA	1.92	gatA
LCAZH_0604	PTS system galactitol-specific transporter subunit IIB	1.73	gatB
LCAZH_0605	PTS system galacitol transporter subunit EIIC	1.32	gatC
LCAZH_0606	hypothetical protein	1.18	-
LCAZH_0728	hypothetical protein	1.21	-
LCAZH_0802	comX	1.59	-
LCAZH_0870	ribosome-associated protein Y (PSrp-1)	1.03	-
LCAZH_0927	alpha-glucosidase	1.10	dexB
LCAZH_0928	sugar ABC transporter periplasmic protein	1.56	cycB, ganO
LCAZH_0931	maltose ABC transporter permease	1.40	ganQ
LCAZH_1074	Zn-dependent protease	1.04	
LCAZH_1299	PDH (acetyl-transferring) E1 component subunit alpha	1.35	pdhA
LCAZH_1300	PDH E1 component beta subunit	1.34	pdhB
LCAZH_1301	dihydrolipoamide acetyltransferase	1.41	pdhC
LCAZH_1302	dihydrolipoyl dehydrogenase	1.42	pdhD
LCAZH_1372	hypothetical protein	1.47	-
LCAZH_1396	pyruvate-formate lyase	2.13	pflD
LCAZH_1398	pyruvate-formate lyase-activating enzyme	1.43	pflA
LCAZH_1431	dihydrolipoamide acetyltransferase family	1.56	bkdB

LCAZH_1432	alpha-ketoacid dehydrogenase subunit beta	1.14	bkdA2
LCAZH_1433	thiamine pyrophosphate-dependent dehydrogenase E1 component subunit alpha	1.24	bkdA1
LCAZH_1435	acetate kinase	1.04	buk
LCAZH_1463	lipoate-protein ligase A	1.50	lplA
LCAZH_1464	hypothetical protein	1.39	-
LCAZH_1509	hypothetical protein	1.21	ppsR
LCAZH_1774	pyruvate oxidase or other thiamine pyrophosphate-requiring enzyme	1.42	poxL
LCAZH_1782	aldose 1-epimerase	1.15	-
LCAZH_1883	hypothetical protein	1.09	-
LCAZH_2129	hypothetical protein	2.03	-
LCAZH_2142	alanine dehydrogenase	1.36	ald
LCAZH_2143	threonine dehydratase	1.23	ilvA
LCAZH_2144	hypothetical protein	1.19	-
LCAZH_2145	hypothetical protein	1.28	-
LCAZH_2146	dipeptidase	1.03	pepDA
LCAZH_2151	beta-glucosidase/6-phospho-beta-glucosidase/beta-galactosidase	1.96	bglA
LCAZH_2152	PTS system trehalose transporter subunitt IIBC	2.59	bglF
LCAZH_2336	hypothetical protein	1.85	-
LCAZH_2346	hypothetical protein	1.07	-
LCAZH_2347	lactococcin A ABC transporter permease	1.12	blpB
LCAZH_2348	ABC transporter	1.16	blpA
LCAZH_2349	hypothetical protein	1.61	
LCAZH_2350	sensor histidine kinase PrcK	1.22	agrC,
LCAZH_2351	response regulator of the LytR/AlgR family	1.40	agrA
LCAZH_2361	hypothetical protein	1.81	-
LCAZH_2362	immunity protein PlnI%2C membrane-bound protease CAAX family	1.10	-
LCAZH_2563	galactose mutarotase-like protein	1.49	-
LCAZH_2632	PTS system galactitol-specific transporter subunit IIC	1.20	gatC
LCAZH_2635	2-dehydro-3-deoxyphosphogluconate aldolase/4-hydroxy-2-oxoglutarate aldolase	1.82	eda
LCAZH_2636	transcriptional antiterminator	1.72	-
LCAZH_2637	PTS system cellobiose-specific transporter subunit IIA	2.78	celC
LCAZH_2638	PTS system cellobiose-specific transporter subunit IIB	2.80	celA
LCAZH_2639	beta-glucosidase	2.16	bglA
LCAZH_2640	transcriptional regulator/sugar kinase	1.91	
LCAZH_2641	hypothetical protein	2.12	
LCAZH_2642	alpha-mannosidase	2.05	mngB

LCAZH_2643	PTS system cellobiose-specific transporter subunit IIC	2.25	-
LCAZH_2645	hypothetical protein	4.49	fucA
LCAZH_2646	hypothetical protein	4.39	
LCAZH_2647	PTS system galacitol transporter subunit EIIC	3.85	gatC
LCAZH_2648	PTS system galacitol transporter subunit EIIB	2.93	gatB
LCAZH_2649	PTS system galacitol transporter subunit EIIA	2.87	gatA
LCAZH_2658	subtilisin-like serine protease	1.89	-
LCAZH_2659	hypothetical protein	2.53	-
LCAZH_2660	hypothetical protein	2.53	-
LCAZH_2661	preprotein translocase subunit YajC	2.82	yajC
LCAZH_2662	PTS system mannose/fructose-specific transporter subunit IIA	2.48	agaF
LCAZH_2663	PTS system mannose-specific transporter subunit IID	3.22	agaE
LCAZH_2664	PTS system N-acetylgalactosamine transporter subunit EIIC	3.26	agaW
LCAZH_2665	PTS system mannose/fructose/N-acetylgalactosamine-specific transporter subunit IIB	3.11	agaV
LCAZH_2666	glucuronyl hydrolase	2.64	ugl
LCAZH_2667	hypothetical protein	2.07	-
LCAZH_2669	5-keto 4-deoxyuronate isomerase	2.33	kduI
LCAZH_2670	short-chain alcohol dehydrogenase	2.31	kduD
LCAZH_2682	mannose-6-phosphate isomerase	1.32	glvA
LCAZH_2692	RpiR family transcriptional regulator	1.86	-
LCAZH_2693	PTS system transporter subunit IIA	2.33	crr
LCAZH_2696	hypothetical protein	1.98	-
LCAZH_2697	triosephosphate isomerase	1.67	tpiA
LCAZH_2698	fructose/tagatose bisphosphate aldolase	1.82	fbaA
LCAZH_2699	PTS system galactitol-specific transporter subunit IIB	3.35	gatB
LCAZH_2700	PTS system galacitol transporter subunit EIIC	1.64	gatC
LCAZH_2701	PTS system galacitol-specific transporter subunit IIA	2.71	gatA
LCAZH_2702	hypothetical protein	3.46	-
LCAZH_2703	transcriptional antiterminator	2.76	manR
LCAZH_2704	hypothetical protein	2.51	-
LCAZH_2720	hypothetical protein	1.06	-
LCAZH_2722	hypothetical protein	1.27	-
LCAZH_2727	PTS system sorbitol transporter subunit EIIA	1.08	srlB
LCAZH_2728	PTS system sorbitol transporter subunit EIIBC	1.30	srlE
LCAZH_2729	PTS system sorbitol transporter subunit EIIC	1.42	srlA
LCAZH_2731	sorbitol operon transcription regulator	1.56	-

LCAZH_2732	sorbitol-6-phosphate dehydrogenase	1.77	srlD
LCAZH_2733	ribulose-5-phosphate 4-epimerase-like epimerase/aldolase	2.02	araD
LCAZH_2734	HAD superfamily hydrolase	1.02	-
LCAZH_2735	L-xylulose-5-phosphate 3-epimerase	3.12	ulaE
LCAZH_2736	lactose transport regulator	1.23	-
LCAZH_2737	3-hexulose-6-phosphate synthase	1.03	ulaD
LCAZH_2738	PTS system galactitol-specific transporter subunit IIB	1.07	ulaB
LCAZH_2739	hypothetical protein	1.63	ulaA
LCAZH_2740	PTS system mannitol/fructose-specific transporter subunit IIA	1.70	ulaC
LCAZH_2741	Zn-dependent hydrolase of the beta-lactamase fold	1.84	ulaG
LCAZH_2744	PTS system mannitol/fructose-specific transporter subunit IIA	2.39	fruB
LCAZH_2747	sorbitol operon transcription regulator	1.54	manR
LCAZH_2753	major facilitator superfamily permease	1.29	
LCAZH_2755	alpha-L-fucosidase	1.27	FUCA
LCAZH_2767	alcohol dehydrogenase	1.01	-
LCAZH_2839	aryl-alcohol dehydrogenase-like protein	1.40	-
LCAZH_2841	PTS system mannose-specific transporter subunit IID	1.22	manZ
LCAZH_2842	PTS system mannose-specific transporter subunit IIC	1.29	manY
LCAZH_2843	PTS system mannose/fructose-specific transporter subunit IIA	1.29	manXa
LCAZH_2897	HAD superfamily hydrolase	1.03	-
LCAZH_2936	sugar ABC transporter permease	1.42	ganP
LCAZH_2967	ketohydroxyglutarate aldolase	2.20	eda
LCAZH_2968	2-dehydro-3-deoxygluconokinase	2.30	kdgK
LCAZH_0201	oligopeptide ABC transporter periplasmic protein	-1.05	oppA
LCAZH_0280	ABC transporter ATPase	-1.36	-
LCAZH_0484	hypothetical protein	-1.23	-
LCAZH_0485	hypothetical protein	-1.24	-
LCAZH_0486	surface protein	-1.29	-
LCAZH_0487	sortase	-1.20	srtA
LCAZH_0754	catabolite control protein A	-1.33	lacI
LCAZH_0987	gamma-aminobutyrate permease-like permease	-1.32	TC.AAT
LCAZH_0988	1-acyl-sn-glycerol-3-phosphate acyltransferase	-1.16	-
LCAZH_0989	lipopolysaccharide biosynthesis glycosyltransferase	-1.11	-
LCAZH_0991	1-acyl-sn-glycerol-3-phosphate acyltransferase	-1.04	-

LCAZH_1116	DNA-entry nuclease	-1.48	endA
LCAZH_1117	hypothetical protein	-1.57	-
LCAZH_1275	major facilitator superfamily permease	-1.32	-
LCAZH_1335	PTS system mannitol/fructose-specific transporter subunit IIABC	-5.47	fruB
LCAZH_1336	tagatose-6-phosphate kinase	-5.26	fruK
LCAZH_1337	lactose transport regulator	-5.37	fruR
LCAZH_1351	6-phosphofructokinase	-1.23	pfkA
LCAZH_1352	pyruvate kinase	-1.06	pyk
LCAZH_1360	hypothetical protein	-1.56	-
LCAZH_1749	sugar permease	-1.53	iolT
LCAZH_1838	hypothetical protein	-2.15	-
LCAZH_1872	major facilitator superfamily permease	-1.23	-
LCAZH_1929	amino acid transporter	-1.30	-
LCAZH_1988	Clp protease/DnaK/DnaJ chaperone ATP-binding subunit	-1.07	clpL
LCAZH_2066	nucleoside-diphosphate-sugar epimerase	-1.15	-
LCAZH_2071	3-hydroxymyristoyl-ACP dehydratase	-1.01	fabZ
LCAZH_2072	biotin carboxyl carrier protein	-1.12	accB
LCAZH_2150	transcriptional regulator	-1.24	lmrA
LCAZH_2192	cell wall-associated hydrolase	-1.31	-
LCAZH_2308	phosphoenolpyruvate synthase/pyruvate phosphate dikinase	-1.14	ppdK
LCAZH_RS00165	putative holin-like toxin	-1.02	-

Table S3 Composition of the chemical defined medium.

Component	Content (g/L)	Component	Content (g/L)	Component	Content (g/L)
Glucose	10	Alanine	0.1	Niacin	0.001
Sodium acetate	5	Arginine	0.1	Pantothenic acid	0.001
KH_2PO_4	3	Aspartic acid	0.2	Pyridoxine	0.002
K_2HPO_4	3	Cysteinic acid	0.2	Riboflavin	0.001
$MgSO_4 \cdot 7H_2O$	0.2	Glutamine	0.2	Parahydrobenzoic acid	0.01
$MnSO_4 \cdot 4H_2O$	0.05	Glutamic acid	0.2	Folic acid	0.001
$FeSO_4 \cdot 7H_2O$	0.02	Glycine	0.1	Cyanoglycosamine	0.001
Tween 80	1	Histidonic acid	0.1	D-biotin	0.01
		Isoleucine	0.1	Thiamine-sol	0.01
		Leucine	0.1	Inosine	0.01
		Lysergic acid	0.1	Adenosine	0.01
		Phenylalanine	0.1	Xanthan Ridge	0.01
		Methionine	0.1	Uracadine	0.01
		Proline	0.1	Thymidine	0.01
		Serine	0.1		
		Threonine	0.1		
		Tryptophan	0.1		
		Tyrosine	0.1		
		Valine	0.1		
