# Supplementary material for: Roles of adenine methylation in the physiology of Lacticaseibacillus paracasei
Source: Nat Commun. 2023 May 6;14:2635. doi: 10.1038/s41467-023-38291-1 (PMC10164179; doi:10.1038/s41467-023-38291-1)
Supplement: Supplementary file 2 — Description of Additional Supplementary Files [file 41467_2023_38291_MOESM2_ESM.pdf]

Description of Additional Supplementary Files:

Supplementary Data 1: Differentially expressed proteins in pglX mutant in comparison with its wild type.

Supplementary Data 2: Altered expression of carbohydrate metabolism-related genes in unique interaction (UI) regions, chromosomal interaction domain (CID) regions, and insulation areas (IAs) in pglX mutant.

Supplementary Data 3: Distribution of transcription factor binding site (TFBS) motifs across 28 Lacticaseibacillus paracasei isolates.

Supplementary Data 4: Information of downstream genes of the conserved motifs.

Supplementary Data 5: Information of primers used in real timequantitative polymerase chain reactions (RT-qPCRs).
