# Supplementary material for: Roles of adenine methylation in the physiology of Lacticaseibacillus paracasei
Source: Nat Commun. 2023 May 6;14:2635. doi: 10.1038/s41467-023-38291-1 (PMC10164179; doi:10.1038/s41467-023-38291-1)
Supplement: Supplementary file 8 — Reporting Summary [file 41467_2023_38291_MOESM8_ESM.pdf]

nature portfolio

Corresponding author(s): Wenyi Zhang

Last updated by author(s): Apr 8, 2023

Reporting Summary

Nature Portfolio wishes to improve the reproducibility of the work that we publish. This form provides structure for consistency and transparency in reporting. For further information on Nature Portfolio policies, see our Editorial Policies and the Editorial Policy Checklist.

Statistics

For all statistical analyses, confirm that the following items are present in the figure legend, table legend, main text, or Methods section.

n/a	Confirmed	
☐	☒	The exact sample size (*n*) for each experimental group/condition, given as a discrete number and unit of measurement
☒	☐	A statement on whether measurements were taken from distinct samples or whether the same sample was measured repeatedly
☐	☒	The statistical test(s) used AND whether they are one- or two-sided<br>*Only common tests should be described solely by name; describe more complex techniques in the Methods section.*
☒	☐	A description of all covariates tested
☐	☒	A description of any assumptions or corrections, such as tests of normality and adjustment for multiple comparisons
☐	☒	A full description of the statistical parameters including central tendency (e.g. means) or other basic estimates (e.g. regression coefficient) AND variation (e.g. standard deviation) or associated estimates of uncertainty (e.g. confidence intervals)
☐	☒	For null hypothesis testing, the test statistic (e.g. *F*, *t*, *r*) with confidence intervals, effect sizes, degrees of freedom and *P* value noted<br>*Give P values as exact values whenever suitable.*
☒	☐	For Bayesian analysis, information on the choice of priors and Markov chain Monte Carlo settings
☒	☐	For hierarchical and complex designs, identification of the appropriate level for tests and full reporting of outcomes
☐	☒	Estimates of effect sizes (e.g. Cohen's *d*, Pearson's *r*), indicating how they were calculated

Our web collection on statistics for biologists contains articles on many of the points above.

Software and code

Policy information about availability of computer code

Data collection	No software was used.
Data analysis	SMRT link v5.1.0, Prokka v1.13, RASTv2.0, MEME suite v5.0.5, WebLogo3, Bowtie2 v2.4.4, BLAST v2.2.26, DESeq2 v1.12.3, DiscoverTM v2.4, Trimmomatic v0.38, Fit-Hi-C v2.0.5, Analyst v1.6.3, Multiquant v3.0.3, R v4.0.5.<br><br>The ANI was calculated by a standalone java ANI calculator. The methylation site calling used SMRT link v9.0.0.92188. The upstream regions were extracted using python script (https://github.com/peterthorpe5/intergenic_regions). Metabolite identification was based on the MetWare online platform (http://www.metware.cn/).

For manuscripts utilizing custom algorithms or software that are central to the research but not yet described in published literature, software must be made available to editors and reviewers. We strongly encourage code deposition in a community repository (e.g. GitHub). See the Nature Portfolio guidelines for submitting code & software for further information.

Data

Policy information about availability of data

All manuscripts must include a data availability statement. This statement should provide the following information, where applicable:

- Accession codes, unique identifiers, or web links for publicly available datasets
- A description of any restrictions on data availability
- For clinical datasets or third party data, please ensure that the statement adheres to our policy

The raw data of L. paracasei strains obtained from Illumina and SMRT sequencing have been deposited in the NCBI Sequence Read Archive (SRA; http://trace.ncbi.nlm.nih.gov/Traces/sra/sra.cgi) under the accession numbers: SRR16925174-SRR16925228. The genome sequence of L. paracasei Zhang was retrieved from NCBI under the accession number CP001084.2. The MS proteomics data have been deposited to the ProteomeXchange Consortium via the PRIDE partner repository (http://www.ebi.ac.uk/pride; data set identifier, PXD026826). The transcriptomic data have been deposited in the NCBI SRA under the accession number PRJNA725355. The Hi-C data of L. paracasei Zhang and its mutant have been deposited in NCBI SRA under the accession number SAMN23078205.

Human research participants

Policy information about studies involving human research participants and Sex and Gender in Research.

Reporting on sex and gender	Not applicable.
Population characteristics	Not applicable.
Recruitment	Not applicable.
Ethics oversight	Not applicable.

Note that full information on the approval of the study protocol must also be provided in the manuscript.

Field-specific reporting

Please select the one below that is the best fit for your research. If you are not sure, read the appropriate sections before making your selection.

☒ Life sciences ☐ Behavioural & social sciences ☐ Ecological, evolutionary & environmental sciences

For a reference copy of the document with all sections, see nature.com/documents/nr-reporting-summary-flat.pdf

Life sciences study design

All studies must disclose on these points even when the disclosure is negative.

Sample size	Twenty-eight L. paracasei isolates and a pglX gene-inactivated strain of L. paracasei Zhang were used in this study. No statistical method was used to predetermine sample size; rather, all isolated strains and the pglX gene-inactivated strain were investigated.
Data exclusions	No data were excluded from the analyses.
Replication	There are no experimental replicates in the study. But, the analysis can be reproduced using the data and software described in the Methods section, since the sequencing data and the genomes are accessible to the public.
Randomization	No randomization was performed. All strains in this study were investigated under the same conditions and did not require random assignment of strains.
Blinding	Investigators were not blinded. Blinding during analysis was not necessary because the results are quantitative and did not require subjective judgment or interpretation. Additionally, blinding was not possible since each experiment was conducted by a single person and sample labeling was required.

Reporting for specific materials, systems and methods

We require information from authors about some types of materials, experimental systems and methods used in many studies. Here, indicate whether each material, system or method listed is relevant to your study. If you are not sure if a list item applies to your research, read the appropriate section before selecting a response.

Materials & experimental systems

n/a	Involved in the study
☒	☐ Antibodies
☒	☐ Eukaryotic cell lines
☒	☐ Palaeontology and archaeology
☒	☐ Animals and other organisms
☒	☐ Clinical data
☒	☐ Dual use research of concern

Methods

n/a	Involved in the study
☒	☐ ChIP-seq
☒	☐ Flow cytometry
☒	☐ MRI-based neuroimaging
